# Genome-wide identification and characterization of argonaute, dicer-like, and RNA-dependent RNA polymerase gene families in potato *(Solanum tuberosum)*: Advancing RNA interference-based crop enhancement

**DOI:** 10.1371/journal.pone.0339021

**Published:** 2026-02-09

**Authors:** Md. Nahid Hasan Shuvo, Mahmudul Hassan, Md. Abu Musa, Riday Hossain, Jesmin Naher Konak, Zobaer Akond, Fee Faysal Ahmed

**Affiliations:** 1 Department of Mathematics, Faculty of Sciences, Jashore University of Science and Technology, Jashore, Bangladesh; 2 Department of Pharmacy, Faculty of Biological Sciences & Technology, Jashore University of Science and Technology, Jashore, Bangladesh; 3 Department of Biochemistry and Molecular Biology, Mawlana Bhashani Science and Technology University, Santosh, Tangail, Bangladesh; 4 Agricultural Economics and Statistics Section, Horticulture Research Center, Bangladesh Agricultural Research Institute, Gazipur, Bangladesh; Banaras Hindu University, INDIA

## Abstract

In eukaryotic species, RNA silencing is a conserved mechanism for controlling gene expression. The RNA-dependent *RNA polymerase (RDR), argonaute (AGO)*, and *dicer-like (DCL)* proteins are essential for RNA silencing. The RNA interference (RNAi) system regulates eukaryotic gene expression throughout growth, development, and stress response. It is also closely linked the post-transcriptional gene silencing (PTGS) process. The potato is one of the four major food crops and a staple meal in the world that has a great potential to combat global malnutrition. However, no genome-wide analysis of the silencing gene family has yet to be conducted in the economically significant plant potato. In this study, we identified 29 (6 *StDCL*, 14 *StAGO* and 9 *StRDR*) candidate genes in potato. These genes correspond to the *Arabidopsis thaliana* RNAi silencing genes. The analysis of the conserved domain, motif, and gene structure for *StDCL*, *StAGO*, and *StRDR* genes showed higher homogeneity within the same gene family. The Gene Ontology (GO) enrichment analysis exhibited that the identified RNAi genes could be involved in RNA silencing and associated metabolic pathways. A number of important transcription factors (TFs), BBR-BPC, bHLH, bZIP, C2H2, Dof, ERF, MIKC MADS, WRKY families, were identified by network and sub-network analyses between TFs and candidate RNAi gene families. Furthermore, the *cis*-acting regulatory elements (CREs) related to light, stress and hormone responsive functions and tissue-specific expression were identified in candidate genes. These genome wide analyses of these RNAi gene families provide valuable information related to RNA silencing which might be helpful for potato improvement in the breeding program.

## 1. Introduction

Gene silencing is an important regulatory mechanism in eukaryotic organisms that is based on small RNAs (sRNAs) of 21–24 nucleotides in length, such as microRNAs (miRNAs) and small interfering RNAs (siRNAs) [1]. Plant genomes harbor a dense and heterogeneous population of small RNA (sRNA) molecules, which are processed from invading RNA molecules, and they have essential functions in gene silencing and regulation [2]. The regulatory mechanisms governing sRNA molecules in plants involve proteins from three primary families: *DCL*, *AGO*, and *RDR*, which interact with various regulatory components to modulate sRNA biogenesis and activity [3]. The genes associated with *DCL*, *AGO*, and *RDR* are vital for RNAi pathways, regulating gene expression and facilitating gene silencing [4]. *DCL* proteins are needed for miRNA and siRNA biogenesis, cutting large double-stranded RNAs into small mature RNAs [5]. Helicase Domain, PAZ Domain, RNase III Domains, and Double-stranded RNA Binding Domain are important functional domains in *DCL* proteins for its activity [6]. *DCL1* is necessary for miRNA biogenesis, processing pri-miRNAs into miRNA/miRNA duplexes [7]. *DCL2*, *DCL3*, and *DCL4* produce siRNAs of specific lengths (22, 24, and 21 nt, respectively) from long perfect dsRNAs [8,9]. Unlike animals and fungi, which have one or two Dicer proteins, plants possess at least four distinct *DCL* proteins with specialized roles in RNA processing [10].

*AGO* proteins are key effectors of RNAi pathways with a fundamental and universal role in gene repression [11]. *AGO* proteins bind to sRNAs to assemble silencing complexes for gene silencing and have gained various functions in different plant species [12]. Identified by the N-terminal, Linker1, PAZ, Linker2, Mid, and PIWI domains, *AGO* proteins are essential to their function in sRNA-guided regulatory pathways, where they mediate mRNA silencing and gene regulation [13].

RNA-dependent RNA polymerases (RdRPs) play key roles in the synthesis of dsRNA from ssRNA, which is further processed to produce siRNAs that play roles in transposable element silencing, DNA methylation, and regulation of plant reproduction and development and breeding programs [14]. RdRp has an endonuclease domain, RdRp domain, and cap-binding domain and is thus a potential candidate for antiviral drug development [15].

Several important plant genes have been found to possess several RNAi-related gene families, such as *DCL*, *AGO*, and *RDR*, using in silico analysis. For example, Strawberries (*Fragaria spp.*) have 13 *AGO*, 6 *DCL*, and 9 *RDR* genes [16]; Sweet orange (*Citrus sinensis*) has 8 *AGO*, 4 *DCL*, and 4 *RDR* genes [17]; Sunflowers (*Helianthus annuus*) contain 20 *AGO*, 5 *DCL*, and 7 *RDR* genes [18]. Quinoa (*Chenopodium quinoa*) contains 9 *AGO*, 5 *DCL*, and 3 *RDR* genes [19]; Onions (*Allium cepa*) have revealed 8 *AGO*, 4 *DCL*, and 4 *RDR* genes through research [20]; Banana (*Musa acuminata*) genome analysis revealed 13 *AGO*, 3 *DCL*, and 5 *RDR* genes [21].

*Arabidopsis thaliana* is also an established model plant species for RNAi research because of its well-characterized genome, ease of genetic manipulation, and the potential to examine a range of plant-microbe interactions that have uncovered sophisticated functions for *DCL* and *AGO* proteins in immunity [22]. Gene Silencing Tools: New gene silencing tools have been established in recent research, which are transforming the understanding of epigenetic regulatory mechanisms. These tools, coupled with *Arabidopsis*’s well-characterized genetics, make it ideal for in silico studies and gene silencing research in plant biology [23]. These findings emphasize the evolutionary conservation and functional diversity of RNAi-related genes across numerous plant species, paving the way for deeper functional studies on gene silencing mechanisms in economically important crops.

Potato (*Solanum tuberosum*) is a crucial staple crop globally, ranking fourth after maize, wheat, and rice [24]. It is a great contributor to national economies and well-being because of its high calorie density and productivity [25]. The increasing market demand has made potatoes an alternative staple that can become a substitute for wheat and rice in certain areas [25]. Potatoes contain high amounts of essential vitamins and minerals like vitamin C, potassium, magnesium, and dietary fiber when eaten with the skin [26]. Additionally, potatoes contain bioactive compounds with antioxidant properties, with growing interest in pigmented cultivars for their potential health benefits [26]. As a possible crop to help end world hunger, sustainable production is essential given that potatoes are highly susceptible to pests and diseases, leading to as much as 70–80%.

The adaptability of *Solanum tuberosum* to environmental stresses is governed by previously identified most of the specialized gene families without gene silencing gene Family. For example, the *GATA* transcription factor family [27], *formin* gene family [28], *Hsp70* gene family [29], *GAox* gene family [30], *Class III peroxidases* gene family [31], *MYB* gene family [32] etc.

Thus, *DCL*, *AGO* and *RDR* gene Family Identification is necessary for Potato to break the constraints of breeders in improving breeding programs. Despite its significance, no *DCL*, *AGO* and *RDR* gene family identification has been conducted on this important crop. Genetic engineering can enhance the breeding process significantly, resulting in improved production and meeting global demand. We have made a start to identify these important gene family members in this economically important plant by using bioinformatics approaches. This study will facilitate genetic enhancements and help humans across the globe by enhancing production and meeting demands. It could reduce expenses for cost, effort, and time. Enhancing genetic resilience through breeding programs requires a deeper understanding of molecular mechanisms underlying stress responses, particularly those mediated by RNAi pathways.

**Fig 1:**
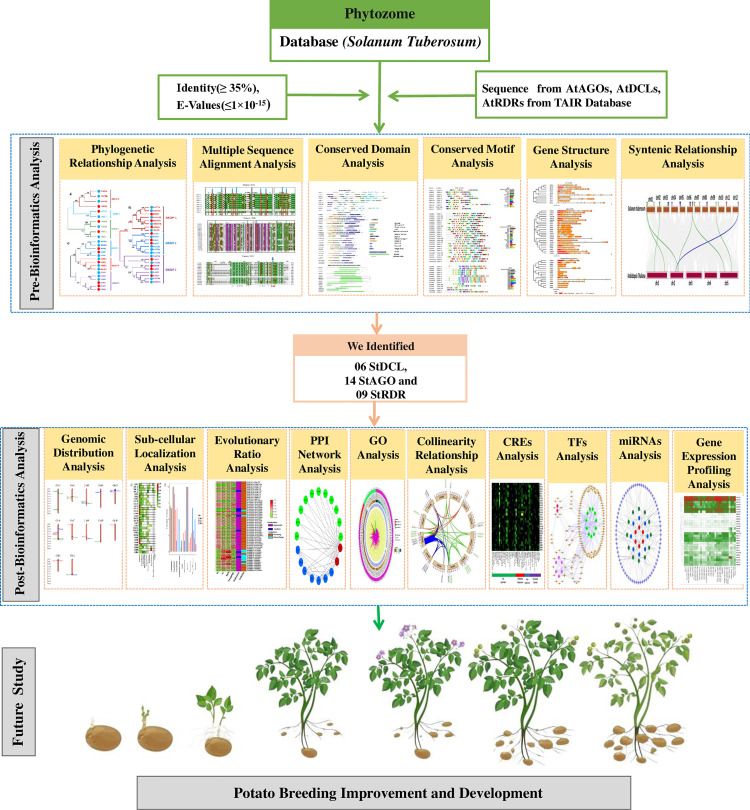
A graphical abstract of our study.

Here, we present the first comprehensive genome-wide identification and analysis of *DCL*, *AGO*, and *RDR* gene families in potato. Using *in silico* approaches, we characterized gene structures, phylogenetic relationships, conserved domains, and chromosomal distributions, drawing comparisons with the model plant *Arabidopsis thaliana*. Further analyses included evaluation of selective pressures (Ka/Ks ratios), synteny, *cis*-regulatory elements (CREs), and protein-protein interaction (PPI) networks. Tissue-specific and drought-induced expression patterns were also investigated to infer functional roles. We have described our study approach graphically in **Fig 1**. This study provides a foundational resource for leveraging RNAi pathways to enhance potato’s agronomic traits, offering insights into evolutionary dynamics and potential targets for biotechnological applications.

## 2. Materials and methods

### 2.1. Data retrieval of *DCL*, *AGO*, and *RDR* genes

To identify *DCL*, *AGO*, and *RDR* genes in *Solanum tuberosum L.*, we retrieved protein sequences from the Phytozome database (v13; https://phytozome.jgi.doe.gov/) using the reference genome of *S. tuberosum* (Assembly: PGSC DM v4.03) [33]. For comparative analysis, reference sequences of *AtDCLs*, *AtAGOs*, and *AtRDRs* from *Arabidopsis thaliana (L.)* were obtained from The *Arabidopsis* Information Resource (TAIR; https://www.arabidopsis.org) [34].

A Hidden Markov Model (HMM)-based Basic Local Alignment Search Tool (BLASTP; https://blast.ncbi.nlm.nih.gov/) search was conducted against the *S. tuberosum* proteome using the customized parameters (E-value ≤1 × 10^-15^, identity ≥ 35%, BLOSUM62 matrix) (**Fig 1**) [35]. Only primary transcripts were retained to avoid redundancy. Genomic coordinates, transcript lengths, and protein sequences were extracted from Phytozome. Gene nomenclature followed phylogenetic clustering with *A. thaliana* orthologs.

### 2.2. Physicochemical characterization

Protein properties including amino acid (AA) length, molecular weight (MW; kDa) and isoelectric point (pI) were predicted using ProtParam (https://web.expasy.org/protparam/) [36]. These parameters provided fundamental insights into protein stability and functional characteristics.

### 2.3. Phylogenetic and sequence alignment analysis

Multiple sequence alignments of *S. tuberosum* and *A. thaliana* proteins were generated using ClustalW (https://www.clustal.org/clustal2/) implemented in Molecular Evolutionary Genetics Analysis (MEGA) version 11.0 [37]. Phylogenetic reconstruction was performed using Neighbor-Joining methods with 1,000 bootstrap replicates [38].

### 2.4. Conserved domain and motif identification

Protein domains were annotated using the Protein family (Pfam; https://pfam.xfam.org) database [39]. Conserved motifs were predicted using Multiple Expectation Maximization for Motif Elicitation (MEME; v5.5.2; http://meme-suite.org/) with parameters set to 6 ≤ width residues ≤ 50 and maximum 20 motifs per protein [40].

### 2.5. Gene structure and chromosomal mapping

Exon-intron structures were analyzed with Gene Structure Display Server (GSDS; v2.0; https://gsds.cbi.pku.edu.cn) [41]. Chromosomal locations were mapped using MapGene2Chromosome (MG2C; v2; http://mg2c.iask.in/) [42], revealing genomic distribution patterns of RNAi pathway genes.

### 2.6. Evolutionary rate (Ka/Ks) analysis

The evolutionary dynamics of duplicated gene pairs, including both segmental and tandem duplications, were analyzed through Ka/Ks (non-synonymous/synonymous substitution rate) analysis. The Ka and Ks values were calculated using MEGA version 11.0 [37]. The Ka/Ks ratio was employed to assess the type of selection pressure acting on each duplicated gene pair [43]. Divergence time for each duplicated gene pair was estimated using the formula *T =* *Ks/(2 × 6.56 × 10*^*−9*^*)* [44], with the resulting values expressed in million years ago (MYA). This analysis enabled the identification of evolutionary pressures and divergence patterns among segmental and tandem duplicated genes in the genome.

### 2.7. Synteny and collinearity analysis

Syntenic blocks between *S. tuberosum* and *A. thaliana* were identified using TBtools (v2.010) [45]. This analysis revealed conserved gene arrangements across evolutionarily diverse species.

### 2.8. PPI network analysis

PPI networks were inferred using Search Tool for the Retrieval of Interacting Genes/Proteins (STRING; v12.0; https://string-db.org) [46] and visualized in Cytoscape (v3.10.0; https://cytoscape.org/) [47]. The networks highlighted potential functional associations among RNAi pathway components.

### 2.9. GO and subcellular localization

GO terms were annotated using Plant Transcription Factor Database (PlantTFDB; http://planttfdb.cbi.pku.edu.cn) [48], providing functional and compartmentalization insights. Subcellular localization of the encoded proteins was predicted using WoLF PSORT (https://wolfpsort.hgc.jp/), a protein localization predictor [49], enabling the determination of their probable cellular compartments and contributing to the understanding of their functional context.

### 2.10. CREs analysis

Promoter regions (2.0 kb upstream of transcription start sites) were scanned for CREs using Plant Cis-acting Regulatory Element (PlantCARE; https://bioinformatics.psb.ugent.be/webtools/plantcare/) database [50]. Identified elements were categorized based on their regulatory functions in plant stress responses and development.

### 2.11. TFs prediction

TFs were predicted with Plant Regulatory Map (PlantRegMap; https://plantregmap.gao-lab.org) using a stringent significance threshold (p-value (1 × 10^−4^)) [51]. The analysis identified potential transcriptional regulators of RNAi pathway genes.

### 2.12. miRNA target prediction

Putative miRNA interactions were identified using plant sRNA target analysis server (psRNATarget; https://www.zhaolab.org/psRNATarget/) [52] with miRNA sequences from miRNA Base (miRBase; v22; https://www.mirbase.org/) [53]. Interaction networks were visualized in Cytoscape (v3.10.0; https://cytoscape.org/) [47].

### 2.13. Comprehensive gene expression profiling across tissues, developmental stages, and stress conditions

RNA-seq data from 29 tissues of potato plants (NCBI BioProject: PRJNA753086, PRJNA489943, PRJNA588378, PRJNA1093478, PRJNA1093480, PRJNA957457, PRJEB52340, PRJNA882516, PRJNA879907 and PRJNA851775) were analyzed using SpudDB Potato Genomics Resource (https://solanaceae.plantbiology.msu.edu/) [54]. Genes with Transcripts Per Million (TPM) > 0.2 were considered expressed, revealing organ-specific expression patterns of RNAi components. The expression patterns were visualized through heatmap generation in TBtools (v2.010) [45], revealing differential expression profiles under Tissues, Developmental Stages, and Stress conditions.

## 3. Results and discussion

### 3.1. *In Silico* identification of RNAi-related genes in potato genome

Using protein sequences of *A. thaliana* (*AtDCL*, *AtAGO*, and *AtRDR*) as query sequences, a HMM was constructed to identify RNA silencing genes in the potato genome. Through this analysis, we identified 6 *DCL (StDCLs)*, 14 *AGO (StAGOs)*, and 9 *RDR (StRDRs)* genes in the potato genome. The basic information of these genes, including chromosomal location, structural features (ORF length, gene length, and intron number), and protein profiles (MW, protein length, and isoelectric point (pI)), is summarized in **Table 1**.

**Table 1:** Basic information of *Solanum tuberosum DCL*, *AGO* and *RDR* gene families.

SL	Gene Name	Accession Number	Start	End	Intron	Extron	Chromosome	ORF (bp)	Protein Length (aa)	MW (kD)	pI
**StDCL**											
**1**	*StDCL1*	Soltu.DM.10G000330.1	266740	288712	20	19	chr10	5745	1915	214.29	6.03
**2**	*StDCL2a*	Soltu.DM.06G011550.1	34920002	34931483	22	21	chr06	4209	1403	158.21	6.41
**3**	*StDCL2b*	Soltu.DM.11G004150.1	4065026	4079168	23	22	chr11	4257	1419	159.92	6.34
**4**	*StDCL2c*	Soltu.DM.11G004160.1	4082208	4101188	42	41	chr11	7443	2481	281.40	6.51
**5**	*StDCL3*	Soltu.DM.08G015780.1	43663284	43691673	18	17	chr08	3093	1031	116.87	6.05
**6**	*StDCL4*	Soltu.DM.07G000050.1	345229	377900	23	22	chr07	4341	1447	162.86	6.57
**StAGO**											
**1**	*StAGO1a*	Soltu.DM.06G027550.1	53125864	53135307	21	20	chr06	3165	1055	117.03	9.46
**2**	*StAGO1b*	Soltu.DM.03G019130.1	43860473	43867957	21	20	chr03	3384	1128	124.70	9.48
**3**	*StAGO3a*	Soltu.DM.02G012290.1	27018909	27023476	3	2	chr02	2466	822	92.68	7.98
**4**	*StAGO3b*	Soltu.DM.02G012280.1	27008740	27015288	3	2	chr02	3156	1052	117.08	9.3
**5**	*StAGO3c*	Soltu.DM.02G012300.1	27031620	27035454	4	3	chr02	3000	1000	112.34	9.18
**6**	*StAGO4a*	Soltu.DM.01G005850.1	6092203	6100362	22	21	chr01	2730	910	101.75	9.01
**7**	*StAGO4b*	Soltu.DM.06G028860.1	54091773	54100564	22	21	chr06	2742	914	101.94	8.95
**8**	*StAGO5*	Soltu.DM.06G030090.1	55171344	55177850	22	21	chr06	3078	1026	114.25	9.39
**9**	*StAGO6a*	Soltu.DM.07G016700.1	46781260	46795060	23	22	chr07	2862	954	107.21	8.58
**10**	*StAGO6b*	Soltu.DM.03G025140.1	50357875	50367374	23	22	chr03	2724	908	102.56	9.49
**11**	*StAGO7*	Soltu.DM.01G010020.1	14102839	14106923	3	2	chr01	3009	1003	114.24	9.2
**12**	*StAGO9*	Soltu.DM.01G035930.1	75445129	75449781	21	20	chr01	2430	810	90.51	9.23
**13**	*StAGO10a*	Soltu.DM.09G025190.1	61128812	61136669	21	20	chr09	2949	983	110.56	9.3
**14**	*StAGO10b*	Soltu.DM.12G024630.1	54556672	54563204	22	21	chr12	2838	946	106.95	9.25
**StRDR**											
**1**	*StRDR1a*	Soltu.DM.05G005560.1	4918024	4928184	4	3	chr05	3348	1116	127.25	8.11
**2**	*StRDR1b*	Soltu.DM.05G005540.1	4888532	4897154	4	3	chr05	3348	1116	126.99	8.3
**3**	*StRDR2*	Soltu.DM.03G028080.1	52675557	52686373	4	3	chr03	3360	1120	127.49	6.51
**4**	*StRDR3*	Soltu.DM.06G009870.1	30119608	30144549	11	10	chr06	1593	531	60.30	8.5
**5**	*StRDR4*	Soltu.DM.12G025310.1	55345495	55358186	20	19	chr12	3333	1111	127.33	7.74
**6**	*StRDR5*	Soltu.DM.06G009880.1	30166824	3017167	7	3	chr06	1065	355	40.24	6.52
**7**	*StRDR6a*	Soltu.DM.08G021290.1	50726283	50728934	2	1	chr08	2196	732	82.96	6.49
**8**	*StRDR6b*	Soltu.DM.04G009340.1	9590937	9595356	2	1	chr04	3516	1172	133.34	7.27
**9**	*StRDR6c*	Soltu.DM.05G004010.1	3609934	3611096	3	2	chr05	636	212	24.69	9.53

6 StDCL genes were identified in the potato genome, with ORF lengths ranging from 3093 bp (*StDCL3*, Soltu.DM.08G015780.1) to 7443 bp (*StDCL2c*, Soltu.DM.11G004160.1). The encoded protein lengths vary from 1031 aa (*StDCL3*) to 2481 aa (*StDCL2c*), with MWs ranging from 116.87 kD (*StDCL3*) to 281.40 kD (*StDCL2c*). The genomic lengths of *StDCL* genes range from 31,672 bp (*StDCL3*) to 42,980 bp (*StDCL4*, Soltu.DM.07G000050.1). All *StDCL* proteins exhibit acidic characteristics, with pI values ranging from 6.03 to 6.57. The acidic isoelectric values (pI) of RNAi pathway proteins (e.g., *AGO*, *DCL*, *RDR*) are critical for their subcellular localization and functional stability, particularly in acidic compartments like the lysosome, where low pI adaptations enhance PPIs and siRNA binding efficiency [55]. Moreover, these pI values guide optimized protein purification strategies, such as ion-exchange chromatography, during in vitro characterization of potato RNAi machinery [56], underscoring their relevance in both functional genomics and biotechnological applications. The presence of conserved domains such as DEAD, Helicase_C, Dicer_dimer, PAZ, RNase III, and DSRM confirms the functional role of *StDCLs* in the RNAi pathway. The acidic pI values of *StDCL* proteins suggest their potential involvement in interactions with other molecules, such as RNA or proteins, in the RNA silencing machinery [57].

A total of 14 *StAGO* genes were identified, with ORF lengths ranging from 2430 bp (*StAGO9*, Soltu.DM.01G035930.1) to 3384 bp (*StAGO1b*, Soltu.DM.03G019130.1). The encoded protein lengths vary from 810 aa (*StAGO9*) to 1128 aa (*StAGO1b*), with MWs ranging from 90.51 kD (*StAGO9*) to 124.70 kD (*StAGO1b*). The genomic lengths of *StAGO* genes range from 4,084 bp (*StAGO9*) to 13,800 bp (*StAGO10a*, Soltu.DM.09G025190.1). All *StAGO* proteins exhibit basic characteristics, with pI values ranging from 7.98 to 9.49. The basic pI values of *StAGO* proteins are consistent with their role in binding sRNAs, such as siRNAs and miRNAs, which are critical for RNA silencing [58]. Interestingly, the variation in ORF lengths, genomic spans, and protein sizes among *StAGOs* implies structural diversity, which may underlie functional divergence. Such diversity has also been reported in other species (e.g., *Arabidopsis thaliana* and *Oryza sativa*), where specific *AGO* clades specialize in distinct RNAi pathways, including miRNA-mediated regulation, tasiRNA biogenesis, and antiviral defense [59]. The larger genomic length of *StAGO10a*, compared with the compact structure of *StAGO9*, may reflect differences in intron content or regulatory elements, potentially influencing transcriptional regulation.

Moreover, the wide range in MWs and AA lengths suggests that certain *StAGOs*, such as *StAGO1b*, could act as central regulators with broader substrate recognition, while smaller *AGOs* like *StAGO9* may have more specialized functions. This interpretation is supported by studies in *Arabidopsis*, where *AGO1* is indispensable for miRNA function and acts broadly across developmental and regulatory processes, whereas *AGO9* is more restricted [60].

9 *StRDR* genes were identified, with ORF lengths ranging from 636 bp (*StRDR6c*, Soltu.DM.05G004010.1) to 3516 bp (*StRDR6b*, Soltu.DM.04G009340.1). The encoded protein lengths vary from 212 aa (*StRDR6c*) to 1172 aa (*StRDR6b*), with MWs ranging from 24.68 kD (*StRDR6c*) to 133.34 kD (*StRDR6b*). The genomic lengths of *StRDR* genes range from 1,162 bp (*StRDR6c*) to 24,941 bp (*StRDR3*, Soltu.DM.06G009870.1). The pI values of *StRDR* proteins range from 6.49 to 9.53, with most proteins exhibiting basic characteristics. The conserved RdRP domain in *StRDR* genes and their basic pI values not only underscore their critical role in amplifying RNAi signals and RNA binding [61,62] but also highlight their evolutionary specialization in potato, where genome-wide diversification of *AGO*, *DCL*, and *RDR* families may fine-tune antiviral defense and gene regulation. However, the potential for off-target silencing due to uncontrolled RdRP activity [63] necessitates further exploration of regulatory mechanisms governing these gene families in *Solanum tuberosum* to optimize RNAi efficiency without compromising cellular homeostasis. The pI values of the identified proteins in potato are consistent with those observed in other plant species, ranging widely from 1.99 to 13.96 [57]. These variations in pI values, from acidic to basic, are critical for post-translational modifications and biochemical interactions within the RNAi machinery [64].

### 3.2. Phylogenetic relationships of RNAi-related genes in *Solanum tuberosum* and *Arabidopsis thaliana*

**Fig 2:**
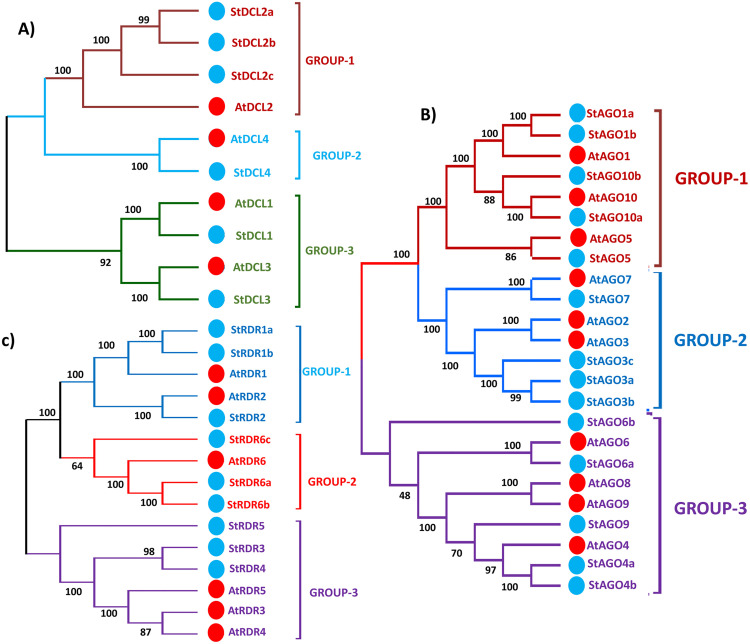
(A–C): Phylogenetic tree for (A) *DCL* proteins (B) *AGO* proteins and (C) *RDR* proteins from *Solanum tuberosum* and *Arabidopsis.* In phylogenetic tree, different groups are represented by different colors; red circles are mentioned genes of *Solanum tuberosum* and green circles are mentioned genes of *A. thaliana*.

By investigation of the phylogenetic relationship between *Solanum tuberosum* and *Arabidopsis thaliana*, we identified total of 29 RNAi-associated genes in potato where 6 are *StDCLs*, 14 are *StAGOs* and 9 are *StRDRs* (**S1****–****S3 Files** **and** **Fig 2A–2C).** The analysis revealed both conserved and divergent evolutionary patterns, offering insights into the functional roles of these proteins in potato.

The *DCL* phylogenetic tree identified three groups (Group I–III) with strong bootstrap support (**Fig 2A**). Group I included *StDCL2a*, *StDCL2b*, and *StDCL2c*, which clustered with *AtDCL2*, confirming their classification into the DCL2 subfamily. These proteins are likely involved in processing long dsRNAs into siRNAs, a critical step in RNA silencing [65]. Group II comprised *StDCL4*, which showed high sequence similarity to *AtDCL4*, suggesting its role in ta-siRNA biogenesis and antiviral defense [66,67]. Such conservation implies that *DCL4* may function as the primary initiator of phased siRNA production, contributing to PTGS and developmental regulation under pathogen challenge. Group III contained *StDCL1* and *StDCL3*, closely related to *AtDCL1* and *AtDCL3*, respectively, implying their involvement in miRNA processing and stress responses [68]. The co-clustering of these isoforms supports the notion that *DCL1*-driven miRNA pathways and *DCL3*-mediated heterochromatic siRNA systems jointly regulate epigenetic homeostasis during stress adaptation.

The *AGO* phylogenetic tree divided the *AGO* genes into three groups (Group I–III) (**Fig 2B**). Group I included *StAGO1a*, *StAGO1b*, *StAGO10a*, *StAGO10b*, and *StAGO5*, which clustered with *AtAGO1*, *AtAGO10*, and *AtAGO5*, respectively. These proteins are likely involved in miRNA-mediated gene silencing, meristem development, and plant growth regulation [69,70]. Their strong evolutionary conservation indicates that these *AGOs* are core effectors in miRNA loading and target slicing, thereby maintaining developmental patterning and tissue differentiation. Group II comprised *StAGO7* and *StAGO3a–c*, closely related to *AtAGO7* and *AtAGO3*, suggesting roles in siRNA-mediated silencing and developmental transitions [71]. This implies that potato *AGO7* homologs could regulate trans-acting siRNAs (ta-siRNA) that coordinate leaf polarity and morphogenesis, similar to mechanisms observed in *Arabidopsis* [72]. Group III included *StAGO6a*, *StAGO6b*, *StAGO9*, *StAGO4a*, and *StAGO4b*, clustering with *AtAGO6*, *AtAGO9*, and *AtAGO4*, respectively. These proteins are predicted to function in epigenetic silencing, reproductive development, and stress responses [73].

The RDR phylogenetic analysis identified three groups (Group I–III) (**Fig 2C**). Group I included *StRDR1a*, *StRDR1b*, and StRDR2, clustering with *AtRDR1* and *AtRDR2*, respectively, suggesting roles in salicylic acid-induced RNA silencing and chromatin modification [74]. Group II comprised *StRDR6a*, *StRDR6b*, and *StRDR6c*, closely related to *AtRDR6*, indicating involvement in ta-siRNA biogenesis and antiviral defense [75]. Group III included *StRDR5*, *StRDR3*, and *StRDR4*, clustering with *AtRDR5*, *AtRDR3*, and *AtRDR4*, respectively, potentially functioning in stress-related RNA silencing [76]. These the presence of multiple stress-responsive *RDR* clades suggests an expanded defensive toolkit in potato, possibly linked to its adaptation to varied environmental stresses and pathogen pressures [72].

The phylogenetic analysis of *DCL*, *AGO*, and *RDR* proteins in *Solanum tuberosum* and *Arabidopsis thaliana* revealed both conserved and divergent evolutionary patterns. The functional predictions for potato proteins, based on their *Arabidopsis* counterparts, align with established roles in RNA silencing, development, and stress responses [65]. The absence of certain genes in the potato genome (*StAGO2*) highlights species-specific adaptations, potentially reflecting unique evolutionary pressures such as pathogen resistance or abiotic stress tolerance [77,78]. Experimental validation—such as gene expression profiling under stress conditions (e.g., viral infection or drought) and functional characterization via CRISPR/Cas9 knockout studies—will be essential to confirm these predictions and elucidate the precise roles of these proteins in potato biology [68,75].

### 3.3. Multiple sequence alignment of *DCL*, *AGO*, and *RDR* proteins in potato and *Arabidopsis*

**Fig 3:**
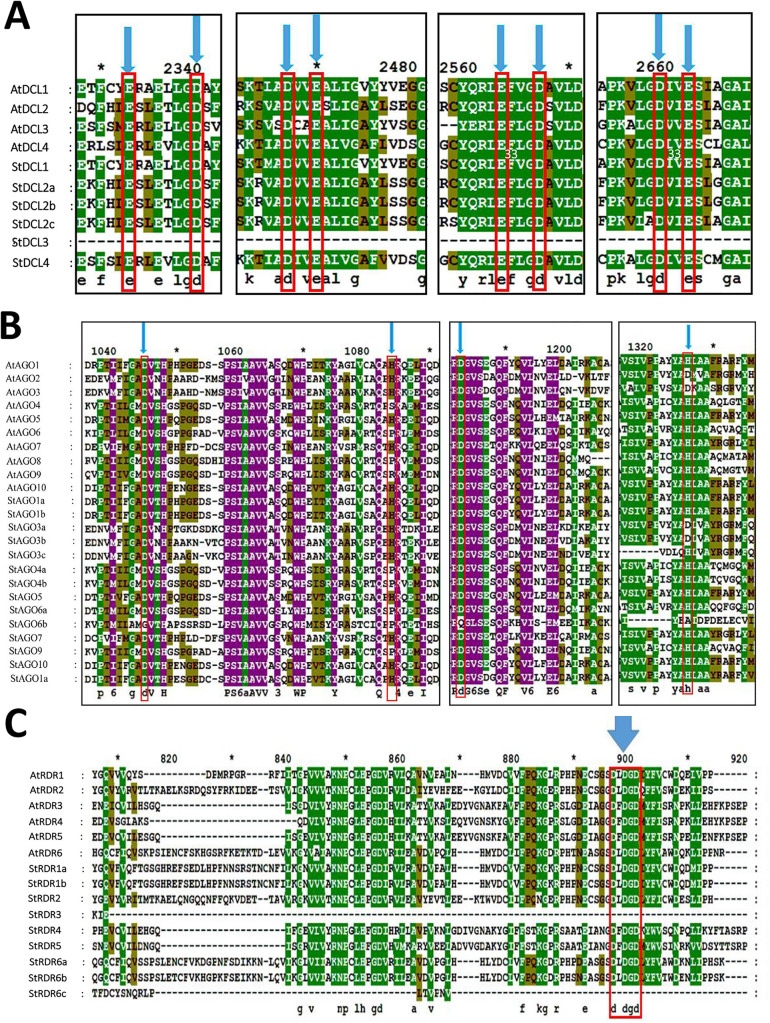
Comparative Multiple Sequence Alignment in *StDCL*, *StAGO*, *StRDR* Gene families. **(A)** Multiple sequence alignment of RNase III domains (RIBOc I and II) from *S. tuberosum* and *Arabidopsis DCL* proteins. Red downward arrows indicate conserved EDDE (glutamate-aspartate-glutamate-aspartate) positions critical for RNase III activity, **(B)** Alignment of PIWI domains from *S. tuberosum* and *Arabidopsis AGO* proteins showing conserved catalytic residues. Red arrows highlight the DDH triad (H798 position indicated) essential for slicer activity, **(C)** RdRP domain alignment of *S. tuberosum* and *Arabidopsis RDR* proteins. The red box encloses the conserved DxDGD catalytic motif required for *RDR* activity.

We performed multiple sequence alignment of the predicted *StDCL*, *StAGO*, and *StRDR* protein sequences with their *Arabidopsis* orthologs (*AtDCL*, *AtAGO*, *AtRDR*) using Clustal-W in MEGA 11 shown at **Fig 3A–3C)**.

Alignment of the RNase III domains revealed strict conservation of the EDDE catalytic motif (glutamate, aspartate, aspartate, glutamate) across all *StDCLs* proteins (*StDCL1*, *StDCL2a*, *StDCL2b*, *StDCL2c*, *StDCL3*, and *StDCL4*) and *AtDCLs* (**Fig 3A)**. Minor variations in flanking regions did not affect the catalytic residues, indicating strong evolutionary pressure to maintain dsRNA-processing activity. This universal conservation highlights the fundamental role of the EDDE motif in initiating the gene silencing cascade by generating the primary sRNA effectors. Notably, *StDCL3* exhibited full conservation of the EDDE motif, unlike *AtDCL3* (**Fig 3A**). This high conservation suggests *StDCLs* retain canonical RNase III functionality, analogous to *Arabidopsis DCLs* in RNAi pathways.

Comparative analysis of the PIWI domains between *Solanum tuberosum* (*StAGO*) and *Arabidopsis thaliana* (*AtAGO*) revealed both conserved catalytic motifs and lineage-specific variations (**Fig 3B**, **Table 2**). The essential DDH/H catalytic triad, required for slicer activity, was fully conserved in *StAGO1a*, *StAGO1b*, *StAGO5*, *StAGO7*, *StAGO10a*, and *StAGO10b* - mirroring their functional counterparts *AtAGO1*, *AtAGO5*, *AtAGO7* and *AtAGO10* in *Arabidopsis*. The preservation of this triad is critical for the effector step of PTGS, enabling the cleavage of target mRNAs. However, several key divergences point to significant functional diversification within the potato *AGO* family.

**Table 2:** Comparison of the *AGO* proteins in PIWI domains between *Solanum tuberosum* and *A. thaliana*.

	*Solanum tuberosum*		*Arabidopsis thaliana*	
SL	AGO	Motif	AGO	Motif
1	*StAGO1a*	DDH/H	*AtAGO1*	DDH/H
2	*StAGO1b*	DDH/H		
3	*StAGO3a*	DDD/H	*AtAGO3*	DDD/H
4	*StAGO3b*	DDD/H		
5	*StAGO3c*	DDH/H		
6	*StAGO4a*	DDH/P	*AtAGO4*	DDH/S
7	*StAGO4b*	DDH/P		
8	*StAGO5*	DDH/H	*AtAGO5*	DDH/H
9	*StAGO6a*	DDH/P	*AtAGO6*	DDH/P
10	*StAGO6b*	GQA/P		
11	*StAGO7*	DDH/H	*AtAGO7*	DDH/H
12	*StAGO9*	DDH/P	*AtAGO9*	DDH/R
13	*StAGO10a*	DDH/H	*AtAGO10*	DDH/H
14	StAGO10b	DDH/H		

^a^Comparison of conserved motif corresponds to D760, D845, H986/H798 of *Arabidopsis* AGO1; D: aspartate, H: histidine, P: proline, R: arginine, S: serine, A: Alanine. G: Glycine and Q: Glutamine

Clade-specific variations were particularly evident in certain subgroups. The *StAGO3a* and *StAGO3b* paralogs feature a DDD/H motif, matching the configuration found in *AtAGO3*, which is known to bind phased siRNAs without exhibiting slicing activity. *StAGO4a* and *StAGO4b* display a DDH/P substitution, differing slightly from *Arabidopsis AtAGO4* (DDH/S), though both variants likely impair slicer activity while maintaining functionality in RNA-directed DNA methylation (RdDM) pathways. These specific substitutions effectively partition the *AGO* family into distinct functional classes, with some members specializing in binding and guiding rather than catalytic cleavage. A particularly striking divergence was observed in *StAGO6b*, which uniquely possesses a GQA/P motif – a configuration not seen in either *AtAGO6* (DDH/P) or other *StAGO* proteins, suggesting potential neofunctionalization in this lineage.

The analysis also revealed notable differences between paralogous genes. While StAGO6a maintains the canonical DDH/H motif, its counterpart *StAGO6b* (GQA/P) and *StAGO9* (DDH/P) show substitutions that may redirect their functional roles toward transcriptional silencing or structural scaffolding functions. These motif variations, summarized in **Table 2**, clearly divide *StAGO* proteins into two functional subgroups: those retaining slicer activity (DDH/H) and those with impaired or altered catalytic potential (DDD/H, DDH/P, GQA/P).

The functional implications of these variations are significant. The DDH/P substitution in *StAGO6b* and *StAGO9* parallels the configuration in *Arabidopsis AtAGO6*, which lacks endonucleolytic activity but participates in RdDM. Similarly, the DDD/H motif in *StAGO3a/b* resembles *AtAGO3*, known to bind siRNAs without cleavage activity. The diversification of catalytic motifs highlights an evolutionary strategy to expand the functional repertoire of the RNA-induced silencing complex (RISC) without gene family expansion. These observations suggest substantial functional specialization within the potato *AGO* family, with *StAGO1a/b* and *StAGO10a/b* likely maintaining their role in PTGS through slicing activity, while variants like *StAGO4a/b* and *StAGO6b* may have evolved specialized functions in transcriptional silencing (TGS) or non-catalytic RNA binding. The unique GQA/P motif in *StAGO6b*, absent in *Arabidopsis*, highlights a potential case of lineage-specific innovation in potato *AGO* evolution. Above results and discursive direction suggest functional specialization within the *AGO* family. While some members likely maintain slicing activity, others may have evolved alternative roles in RdDM [79]. This functional partitioning allows a single protein family to regulate gene expression at multiple levels, from transcript degradation to epigenetic modification. Future transient expression assays could help elucidate how these variations impact RNA silencing efficiency in planta.

Alignment of *StRDR* and *AtRDR* sequences confirmed strict conservation of the DxDGD motif in *StRDR1a*, *StRDR1b*, *StRDR2*, *StRDR5*, and *StRDR6a*, *StRDR6b*, *StRDR6c*, (**Fig 3C**), critical for *RDR* activity. This motif’s conservation is fundamental for amplifying the RNAi signal, as it enables the synthesis of double-stranded RNA from single-stranded templates, thereby propagating the silencing response. Truncations near the catalytic site in *StRDR3* and *StRDR6c* (likely due to incomplete gene models) require cDNA sequencing for resolution. The widespread DxDGD conservation suggests *StRDRs* retain robust dsRNA synthesis roles, similar to *AtRDRs*, in siRNA amplification and antiviral defense.

The DDH/H triad, essential for *in vitro* endonuclease activity in *AtAGOs* [80–82], is disrupted in several *StAGOs*, potentially altering RNA silencing mechanisms. While *StAGO1a* and *StAGO1b* likely retain slicing activity, substitutions in *StAGO4a* and *StAGO4b* and *StAGO6a* may redirect their roles to transcriptional gene silencing (TGS). The strategic alteration of catalytic residues represents a mechanism for functional specialization within the gene silencing machinery, allowing different *AGO* paralogs to undertake distinct regulatory tasks.

### 3.4. Conserved domain analysis of RNAi-related genes in potato and *Arabidopsis*

**Fig 4:**
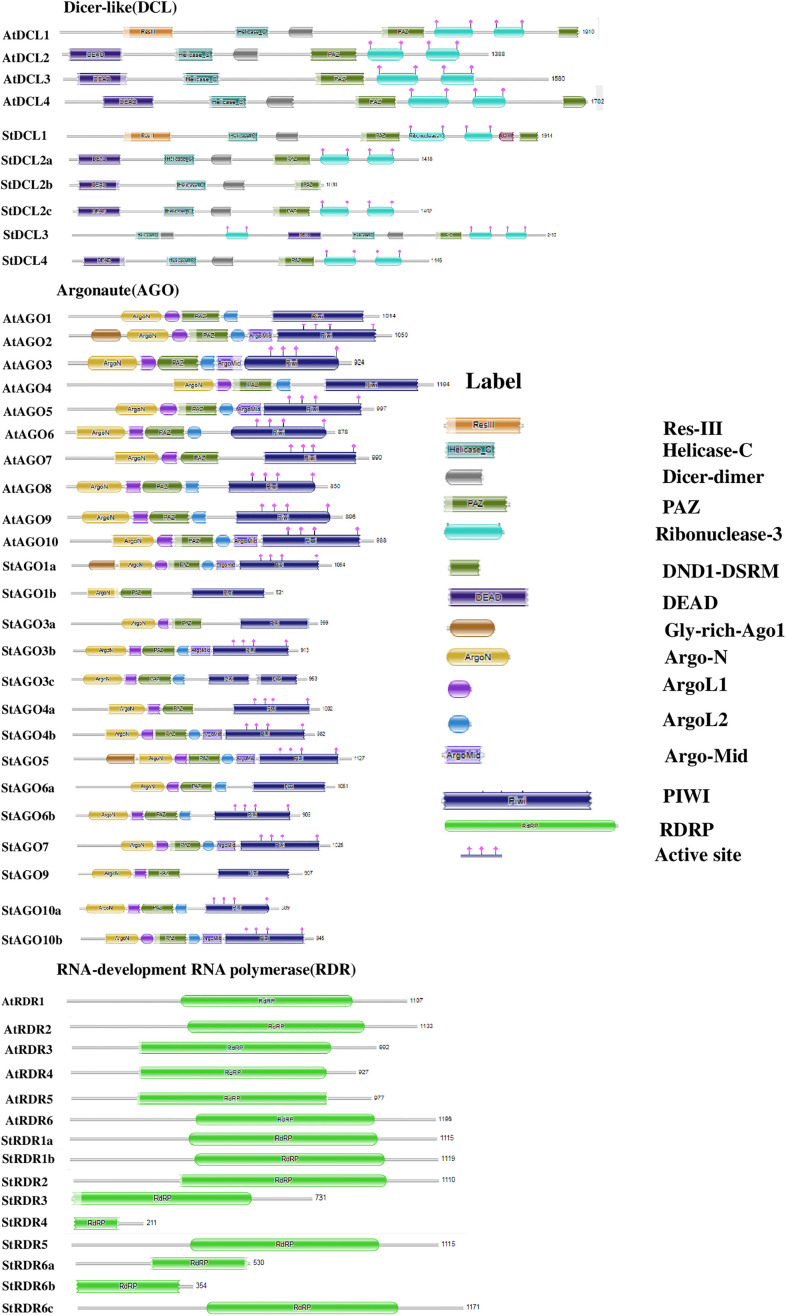
The conserved domains of the predicted *DCL*, *AGO*, and *RDR* proteins in Potato and *Arabidopsis.*

The analysis of conserved domains in RNAi-related gene families (*DCL*, *AGO*, and *RDR*) in potato and *Arabidopsis* provides valuable insights into the evolutionary conservation and functional diversification of these proteins. We identified conserved domains in *StDCL*, *StAGO*, and *StRDR* gene families and compared them with their *Arabidopsis* counterparts shown at **Fig 4**.

The *DCL* proteins family in potato revealed significant conservation of functional domains compared to *Arabidopsis*. *StDCL1*, *StDCL2a*, *StDCL2c*, and *StDCL4* contain all essential domains, including Res-III, Helicase-C, Dicer-dimer, PAZ, Ribonuclease-3, and DND1-DSRM, mirroring their *Arabidopsis* counterparts [83,84]. The presence of these core domains confirms their fundamental role in the initiation of the gene silencing pathway by processing double-stranded RNA into siRNAs. However, *StDCL2b* lacks the Ribonuclease-3 domain, which may impair its endonuclease activity. This absence could result from evolutionary divergence or adaptation to specific stressors in potato, as Ribonuclease-3 is critical for cleaving dsRNA during RNAi [68,85]. The loss of this catalytic domain suggests *StDCL2b* may have a specialized, non-canonical role or requires interaction with other proteins to function in silencing. Additionally, *StDCL3* exhibits a rearranged domain order compared to *AtDCL3*, indicating potential functional novelty. This rearrangement might reflect species-specific adaptations, possibly related to potato’s unique developmental processes or environmental challenges [68,86]. Previous studies have highlighted the role of *DCL* proteins in plant defense against viral infections, and the conservation of these domains in potato underscores their importance in RNAi pathways [84,87]. However, the structural variations observed in *StDCL2b* and *StDCL3* warrant further investigation to elucidate their functional implications through targeted mutagenesis and complementation assays in model systems [88].

The *AGO* family in potato displays a high degree of domain conservation with *Arabidopsis*, particularly in the PAZ and PIWI domains, which are essential for RNA binding and RNase activity [70,71]. All *StAGO* proteins retain these domains, emphasizing their conserved role in RNA silencing. These domains are indispensable for the effector phase of silencing, as the PAZ domain anchors the sRNA while the PIWI domain provides the ‘slicer’ activity for target cleavage. In contrast, *StAGO1b* lacks the Gly-rich Ago1 domain, suggesting functional divergence or specialization within the *AGO* family [77,89]. Furthermore, *StAGO3b*, *StAGO4b*, *StAGO5*, *StAGO7*, and *StAGO10b* contain the Argo-Mid domain, which is critical for sRNA binding. However, this domain is absent in several other *StAGO* proteins, indicating potential functional specialization [73,90]. The differential presence of the Argo-Mid domain likely influences the specificity and affinity for different classes of sRNAs, thereby directing distinct silencing outcomes. The diversity in domain composition among *StAGO* proteins may reflect their involvement in distinct biological processes, such as stress responses, developmental regulation, or pathogen defense [69,91]. For instance, the presence of the Argo-Mid domain in *StAGO5* and *StAGO7* suggests their role in gene regulation, while its absence in other *StAGO* variants may indicate redundancy or alternative regulatory mechanisms [92]. These findings highlight the functional complexity of the *AGO* family in potato and underscore the need for further research to characterize their roles in RNAi pathways through comprehensive PPI studies and tissue-specific expression analyses [82,93].

*RDR* proteins in potato exhibit a high degree of conservation in the RdRP domain, similar to their *Arabidopsis* counterparts [74,78]. All *StRDR* proteins contain this domain, which is essential for synthesizing dsRNAs from ssRNAs, a critical step in RNAi silencing. This domain is fundamental for amplifying the RNAi response by creating secondary double-stranded RNA substrates, which reinforces and sustains the gene silencing signal. However, shorter isoforms may represent truncated variants or products of alternative splicing [75,94]. These shorter isoforms could play regulatory roles or act as dominant-negative inhibitors of RNAi pathways [76,95]. Such truncated variants could modulate the efficiency of the silencing pathway by competing with full-length *RDRs*, thus providing a potential layer of regulation. The presence of multiple *RDR* isoforms in potato suggests functional diversification, possibly related to stress responses, viral defense, or developmental regulation [96,97]. Previous studies have shown that *RDR* proteins are essential for amplifying RNAi signals, and their conservation in potato underscores their importance in RNAi-mediated gene regulation [98,99]. However, the functional significance of the shorter isoforms requires further investigation to determine their roles in potato biology through detailed biochemical characterization and genetic complementation experiments [100,101].

### 3.5. Conserved motif analysis of RNAi-related genes in potato and *Arabidopsis*

**Fig 5:**
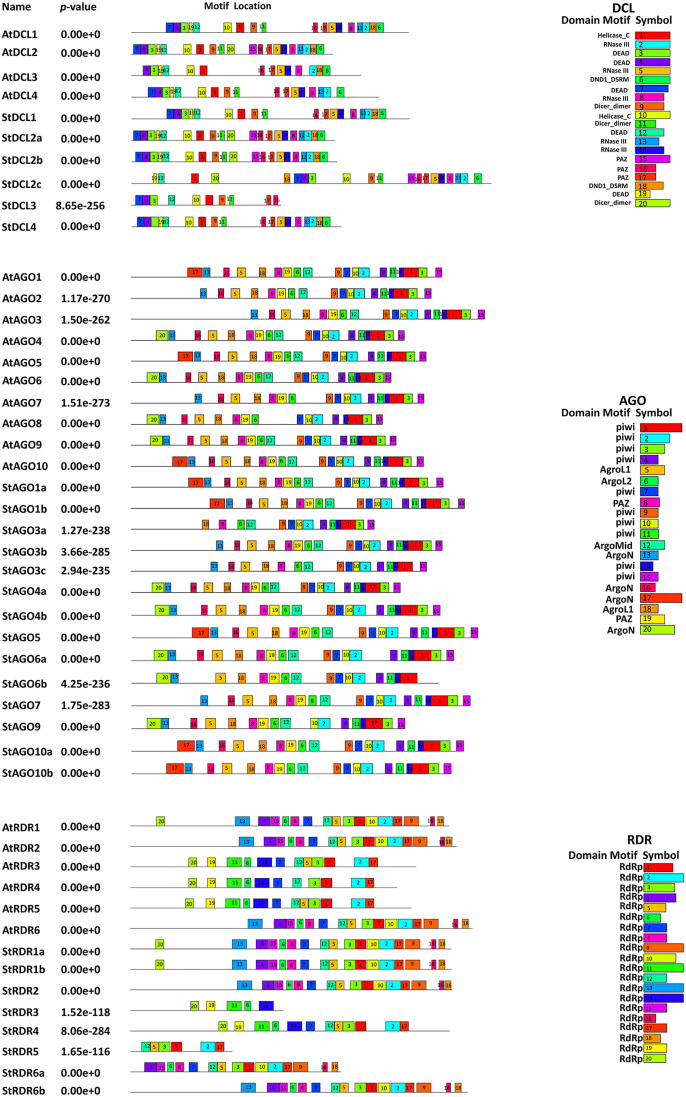
The conserved motifs of the predicted *DCL*, *AGO* and *RDR* protein families in Potato and *Arabidopsis.* Each color represents different motifs in the predicted proteins domains.

We identified conserved motifs in the *DCL*, *AGO*, and *RDR* protein families of *Solanum tuberosum (St)* and compared them with their *Arabidopsis thaliana* (*At*) homologs which shown at **Fig 5**. The analysis revealed both conserved and divergent motifs, providing insights into the structural and functional similarities and differences between the two species.

In the *DCL* family, we identified up to 20 conserved motifs in both *S. tuberosum* and *A. thaliana* proteins. *StDCL1*, *StDCL2a*, *StDCL2b*, and *StDCL4* exhibited complete conservation of 18–20 motifs with their *Arabidopsis* counterparts, *AtDCL1* and *AtDCL4*, indicating strong functional similarity [83,87]. For instance, *StDCL1* and *StDCL4* shared identical motif arrangements with *AtDCL1* and *AtDCL4*, respectively, suggesting that these proteins likely perform similar roles in the RNAi pathway [84,85]. This high degree of conservation underscores their fundamental and non-redundant roles in initiating the gene silencing process by processing double-stranded RNA precursors into sRNAs. However, *StDCL3* displayed only 10 motifs, significantly fewer than *AtDCL3*, which has 14 motifs. Notably, *StDCL3* lacked motifs 19 (DEAD) and 20 (Dicer_dimer), which are present in *AtDCL3* [86,102]. This absence may indicate structural or functional differences in the RNAi machinery of potato compared to *Arabidopsis* [68]. The high conservation of motifs in *StDCL1*, *StDCL2*, and *StDCL4* suggests that these proteins are likely essential for RNAi pathways in potato, similar to their roles in *Arabidopsis* [65]. The DEAD and Dicer_dimer domains are critical for RNA processing and cleavage, and their absence in *StDCL3* may imply a reduced or specialized role in potato [102]. The absence of these core motifs suggests a potential divergence in the molecular mechanism of *StDCL3*, which could influence the biogenesis or function of specific sRNA classes. This could result in altered RNAi efficiency or specificity, potentially affecting the processing of sRNAs [84]. Further experimental validation is needed to determine whether *StDCL3* has a unique function in potato or if it represents a functional divergence from *Arabidopsis DCL* proteins.

In the *AGO* family, we observed a maximum of 20 conserved motifs in both species. *StAGO1a*, *StAGO1b*, *StAGO4a*, *StAGO4b*, *StAGO5*, *StAGO6a*, *StAGO9*, and *StAGO10a/b* showed complete conservation of 18–20 motifs with their *Arabidopsis* homologs, indicating strong functional conservation [70,71]. For example, *StAGO1a* and *StAGO1b* shared identical motif arrangements with *AtAGO1*, suggesting that these proteins likely retain similar RNAi functions [89]. The conserved motifs in these *AGO* proteins are critical for forming the core of the RISC, which is directly responsible for the effector step of silencing through mRNA cleavage or translational repression. However, *StAGO3a* and *StAGO3c* exhibited fewer motifs (14 and 15, respectively) compared to *AtAGO3* (18 motifs). Specifically, *StAGO3a* lacked motifs 13 (ArgoN) and 16 (ArgoN), which are critical for *AGO* protein function [73,90]. This may result in altered RNAi silencing efficiency or specificity in potato [77]. Additionally, *StAGO6b* and *StAGO7* showed slight motif variations, with *StAGO6b* missing motif 3 (Piwi) and *StAGO7* missing motif 12 (ArgoMid) [92]. The high conservation of motifs in *StAGO1*, *StAGO4*, *StAGO5*, and *StAGO10* suggests that these proteins are likely essential for RNAi pathways in potato, similar to their roles in *Arabidopsis* [70]. The ArgoN domain is crucial for sRNA binding, and its absence in *StAGO3a* may impair the protein’s ability to bind target RNAs effectively [89]. Similarly, the missing Piwi and ArgoMid domains in *StAGO6b* and *StAGO7* could affect their catalytic activity or interaction with other RNAi machinery components [73]. These specific motif losses could directly compromise the efficiency of RISC assembly and its gene silencing activity. These variations may indicate species-specific adaptations in the RNAi pathway, potentially influencing gene silencing efficiency or target specificity [90]. Experimental studies are needed to confirm whether these motif variations lead to functional differences in potato *AGO* proteins [77].

In the *RDR* family, we predicted 5–17 conserved motifs in *S. tuberosum* proteins. *StRDR1a*, *StRDR1b*, and *StRDR2* showed complete conservation of 17 motifs with *AtRDR1* and *AtRDR2*, indicating strong functional similarity [74,78]. Similarly, St This conservation highlights their essential role in amplifying the silencing signal by synthesizing double-stranded RNA, which serves as a substrate for *DCL* proteins to generate secondary sRNAs. *RDR6a* and *StRDR6b* exhibited high conservation with *AtRDR6*, suggesting similar roles in RNAi pathways [75]. However, StRDR3 and StRDR5 displayed significantly fewer motifs (5 and 6, respectively) compared to *AtRDR3* (12 motifs). This suggests potential functional divergence or reduced activity in potato [96]. For instance, *StRDR3* lacked motifs 11 (RdRP) and 14 (RdRP), which are present in *AtRDR3* [76]. Additionally, *StRDR4*, while retaining 12 motifs, showed a unique arrangement, which may indicate functional specialization [95]. The high conservation of motifs in *StRDR1*, *StRDR2*, and *StRDR6* suggests that these proteins are likely essential for RNAi pathways in potato, similar to their roles in *Arabidopsis* [74]. The RdRP domain is critical for *RDR* activity, and its absence in *StRDR3* and *StRDR5* may impair their ability to synthesize double-stranded RNA, a key step in the RNAi pathway [75]. This could result in reduced RNAi efficiency or altered target specificity in potato [96]. The unique motif arrangement in *StRDR4* may indicate functional specialization, potentially enabling it to perform a unique role in potato RNAi pathways [100]. Further experimental studies are needed to explore the functional significance of these motif variations and their impact on RNAi mechanisms in potato [101].

### 3.6. Comparative analysis of gene structure in *DCL*, *AGO*, and *RDR* genes in potato and *Arabidopsis*

**Fig 6:**
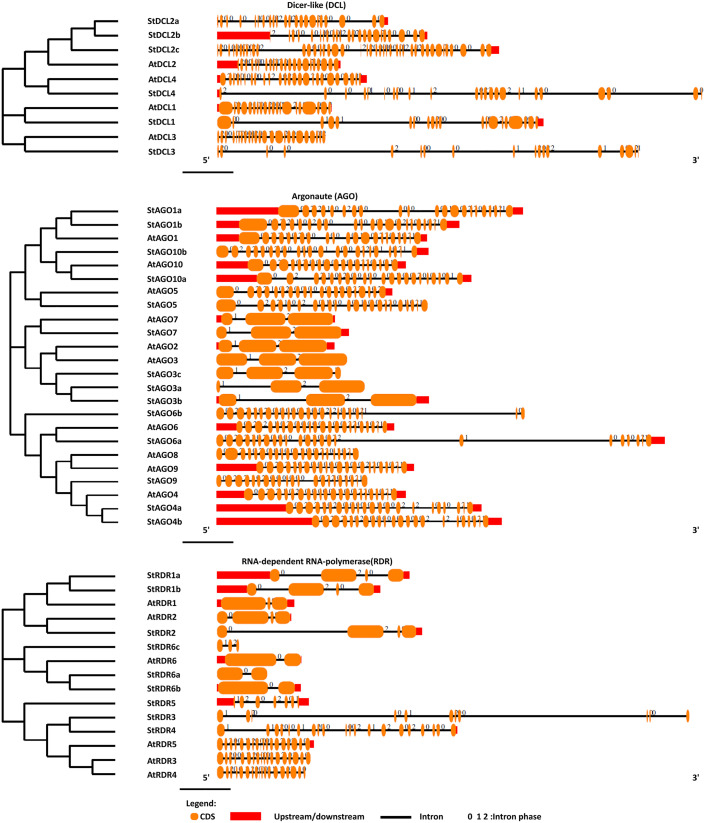
Gene formation of the predicted *DCL*, *AGO*, and *RDR* proteins in *S. tuberosum* with *Arabidopsis.*

The gene structure analysis of *StDCL*, *StAGO*, and *StRDR* genes revealed a high degree of conservation with their orthologs in *Arabidopsis thaliana* (**Fig 6).** The exon-intron organization of these genes demonstrated structural similarities, suggesting functional conservation in the RNAi pathway.

The *StDCL* genes exhibited intron numbers comparable to their *Arabidopsis* counterparts. *StDCL2a* and *StDCL2b* contained 21 and 22 introns, respectively, closely aligning with *AtDCL2* (21 introns). *StDCL4* (22 introns) was structurally similar to *AtDCL4* (24 introns), while *StDCL1* and *AtDCL1* both shared 19 introns. However, *StDCL3* (17 introns) deviated from *AtDCL3* (23 introns), indicating potential functional divergence. *StDCL2c*, with 41 introns, represented an outlier with significantly more introns than its *Arabidopsis* orthologs [68,102]. The presence of this unusually high intron number may contribute to alternative splicing events, potentially generating isoforms with distinct regulatory or stress-adaptive functions. Such intron expansion is often associated with increased transcriptomic plasticity in higher plants for adjusting to abiotic and biotic stresses [103]

Among *StAGO* genes, 12 out of 15 exhibited 19–22 introns, mirroring *Arabidopsis* homologs. For example, *StAGO1a*, *StAGO1b*, and *AtAGO1* all contained 20 introns, while *StAGO10b* (21 introns) resembled *AtAGO10* (18 introns). Exceptions included *StAGO3a*, *StAGO3b*, and *StAGO7*, which had 2, 2, and 3 introns, respectively, similar to *AtAGO2* and *AtAGO7*. *StAGO3c* (3 introns) also deviated slightly from the typical intron range [104,105]. This pattern suggests that *AGO* genes with fewer introns, such as *AGO2* and *AGO7* orthologs, may have undergone intron loss to optimize rapid transcriptional responses during pathogen attack or developmental transitions. Reduced intron load is a known evolutionary strategy to enhance gene expression speed under stress [106,107].

*StRDR* genes showed conserved intron-exon structures, with most containing 2–10 introns. *StRDR1a* and *StRDR1b* (3 introns) matched *AtRDR1* (2 introns) and *AtRDR2* (3 introns). *StRDR6a* and *StRDR6b* (1 intron) mirrored *AtRDR6*, while *StRDR6c* (2 introns) showed minor variation. *StRDR5* (6 introns) and *StRDR3/StRDR4* (10 and 19 introns, respectively) aligned structurally with *AtRDR5* (16 introns) and *AtRDR3/AtRDR4* (17 and 16 introns) [78]. The conservation across *RDR* families underscores their stable evolutionary roles in RNA-dependent amplification of silencing signals. However, intron variation in *StRDR3* and *StRDR5* might indicate selective adaptation of RNAi components to potato-specific stress signaling pathways or virus-host interactions [108].

The structural conservation of *StDCL*, *StAGO*, and *StRDR* genes with their *Arabidopsis* counterparts underscores evolutionary conservation of RNAi pathways. The similarity in intron numbers between *StDCL1*, *StDCL2* and *AtDCL1*, *AtDCL2* supports their roles in miRNA and siRNA biogenesis, respectively [66,102]. The reduced intron count in *StDCL3* and *StAGO3c* may reflect functional divergence, such as specialized stress-response roles [68,104]. Such divergence could provide evolutionary advantages by enabling faster transcription or alternative exon usage under abiotic and biotic stress.

The minimal introns in *StAGO2a*, *StAGO2b* and *StAGO7* mirror *AtAGO2*, *AtAGO7* respectively, which are critical for antiviral defense and developmental regulation [104]. Similarly, the conserved structure of *StRDR1*, *StRDR6* with *AtRDR1*, *AtRDR6* highlights their conserved roles in amplifying RNAi signals [78]. However, structural variations in *StRDR3*, *StRDR5* suggest functional adaptations, potentially fine-tuning RNAi efficiency in potato-specific contexts [105].

These findings align with studies showing that intron-exon architecture influences RNAi machinery efficiency [65]. Overall, the observed structural dynamics reflect the evolutionary optimization of RNAi gene families, balancing stability with adaptability to ensure precise regulation of gene expression in diverse environmental and developmental conditions. Future work should explore how structural variations impact splicing efficiency, protein diversity, and stress adaptation in potato.

### 3.7. Genomic distribution of *StDCL*, *StAGO*, and *StRDR* genes

**Fig 7:**
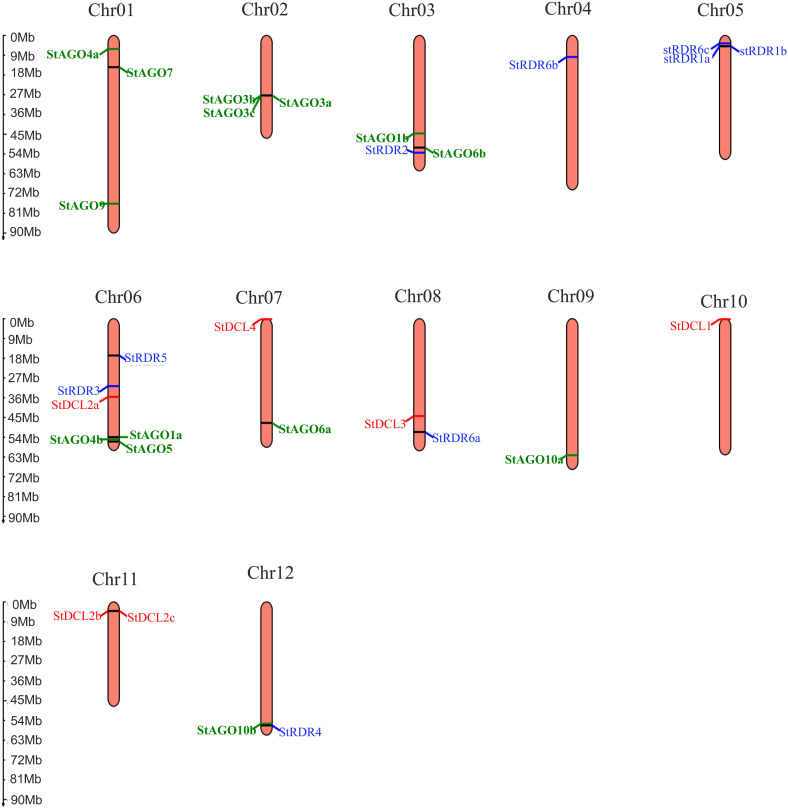
The genomic location of the predicted *StDCL*, *StAGO*,and *StRDR* genes. The scale to indicate the chromosomal length is provided on the left.

The chromosomal mapping of RNAi-related genes in *Solanum tuberosum* revealed non-random distribution patterns across 12 chromosomes (**Fig 7**).

*StDCL* genes localized to chromosomes 6 (*StDCL2a*), 7 (*StDCL4*), 8 (*StDCL3*), 10 (*StDCL1*), and 11 (*StDCL2b*, *StDCL2c*), with notable clustering of *StDCL2b* and *StDCL2c* on chromosome 11 (positions 41.3 Mb and 42.1 Mb respectively). Duplication refers to a genetic mutation where a segment of DNA is replicated one or more times, leading to an increase in the amount of genetic material in the genome. Such duplications can lead to functional diversification or sub-functionalization of paralogs, as observed in other plant *DCL* families [109,110].

*StAGO* genes exhibited broader dispersion, occupying chromosomes 1 (*StAGO4a*, *StAGO7*, *StAGO9*), 2 (*StAGO3a*, *StAGO3b*, *StAGO3c*), 3 (*StAGO1b*, *StAGO6b*), 6 (*StAGO1a*, *StAGO4b*, *StAGO5*), 7 (*StAGO6a*), 9 (*StAGO10a*), and 12 (*StAGO10b*). Three gene clusters were identified: (i) a 1.8 Mb segment on chromosome 1 containing *StAGO4a*, *StAGO7*, and *StAGO9*; (ii) a 3.2 Mb region on chromosome 2 with three *StAGO3* paralogs; and (iii) a 5.6 Mb segment on chromosome 6 harboring *StAGO1a*, *StAGO4b*, and *StAGO5*. Such clusters may facilitate coordinated regulation during stress responses [111].

*StRDR* genes primarily resided on chromosomes 3 (*StRDR2*), 4 (*StRDR6b*), 5 (*StRDR1a*, *StRDR1b*, *StRDR6c*), 6 (*StRDR3*, *StRDR5*), 8 (*StRDR6a*), and 12 (*StRDR4*). This chromosomal distribution pattern suggests both localized clustering and dispersed organization, reflecting the influence of tandem and segmental duplication events in the evolution of the *RDR* gene family. The close proximity of *StRDR1a* and *StRDR1b* on chromosome 5 implies recent duplication, a phenomenon frequently observed in *RDRs* of other *Solanaceae* species such as *Solanum lycopersicum* and *Nicotiana benthamiana*, where duplicated *RDR1* and *RDR6* members have undergone functional diversification to participate in antiviral defense and ta-siRNA biogenesis [14,112].

The scattered localization of *StRDR2*, *StRDR3*, and *StRDR4* across separate chromosomes points toward sub-functionalization or condition-specific expression, consistent with findings from *Arabidopsis thaliana* and *Oryza sativa*, in which *RDR* paralogs display differential regulation under stress and developmental cues [113,114]. Such spatial organization may enhance the functional resilience of the potato RNA silencing machinery, ensuring redundancy and flexibility in response to diverse biotic and abiotic challenges. Collectively, the observed genomic pattern of *StRDRs* highlights the dynamic evolutionary trajectory of the potato RNAi system, maintaining a balance between conserved core function and adaptive specialization.

### 3.8. Sub-cellular localization of RNAi-related genes in potato

**Fig 8:**
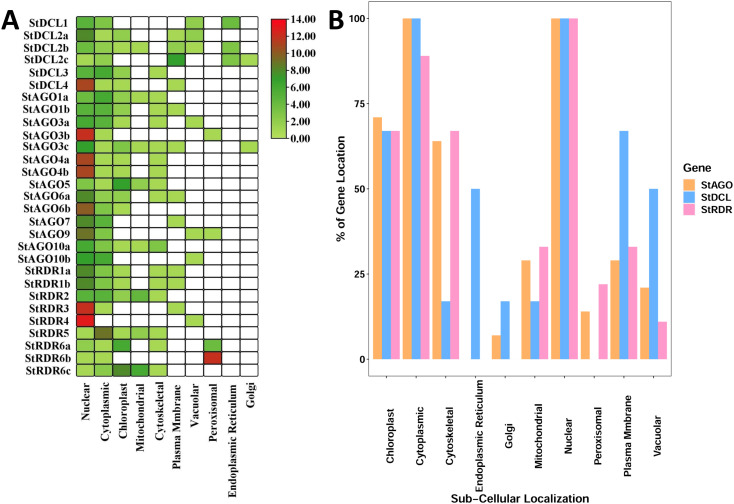
Sub-cellular localization analysis for the (A) *StDCL*, *StAGO*, and *StRDR* proteins. **(B)** The percentage of protein appeared in different cellular organelles.

The subcellular localization of specific proteins is intricately connected to the biological functions of eukaryotic cells. The cellular positioning of these proteins offers valuable insights into their roles and activities within the cell, enabling a deeper understanding of the processes [115,116]. The detected *StDCL*, *StAGO*, and *StRDR* proteins were found in various subcellular compartments, including the nucleus, cytoplasm, chloroplast, mitochondria, cytoskeleton, plasma membrane, vacuole, peroxisome, endoplasmic reticulum, and Golgi apparatus which are shown at (**Fig 8A and Fig 8B**).

The *StDCL* proteins were distributed across multiple compartments, with all six *StDCL* proteins localized in the nucleus and cytoplasm, 66.67% in the chloroplast, 16.67% in the mitochondria, 16.67% in the cytoskeleton, 66.67% in the plasma membrane, 50% in the vacuole, and 50% in the endoplasmic reticulum. Notably, no *StDCL* proteins were found in peroxisomes, while 16.67% were localized in the Golgi apparatus. All *StAGO* proteins (14/14) were found in the nucleus and cytoplasm, with 71.43% localized in the chloroplast, 28.57% in the mitochondria, 64.29% in the cytoskeleton, 28.57% in the plasma membrane, 21.43% in the vacuole, and 14.29% in peroxisomes. No *StAGO* proteins were detected in the endoplasmic reticulum, while 7.14% were found in the Golgi apparatus. Similarly, all *StRDR* proteins (9/9) were localized in the nucleus, with 88.89% in the cytoplasm, 66.67% in the chloroplast, 33.33% in the mitochondria, 66.67% in the cytoskeleton, 33.33% in the plasma membrane, 11.11% in the vacuole, and 22.22% in peroxisomes. No *StRDR* proteins were found in the endoplasmic reticulum or Golgi apparatus.

Earlier research has indicated that RNAi proteins, such as *AGO4* and *DCL3* in *Arabidopsis*, are co-located in the nucleus and play a central role in the RNAi silencing process [117]. The multi-organellar localization of *AGO*, *DCL*, and *RDR* proteins, as evidenced by their presence in nuclei (gene silencing), cytoplasm (mRNA decay), and chloroplasts/mitochondria (organellar gene regulation), underscores their pleiotropic roles in potato RNAi pathways, mirroring findings in other systems where such proteins integrate oxidative phosphorylation, RNA processing, and stress responses [115,116]. However, the functional divergence of these proteins in potato particularly their potential roles in organelle-specific signaling or cytoskeletal RNA transport remains speculative without experimental validation of their spatiotemporal dynamics under biotic/abiotic stresses.

This widespread and overlapping distribution of *StDCL*, *StAGO*, and *StRDR* proteins across multiple subcellular compartments underscores their coordinated involvement in both transcriptional gene silencing (TGS) and PTGS pathways, wherein nuclear-localized components may participate in RdDM and chromatin remodeling, while cytoplasmic and organellar localizations facilitate siRNA biogenesis, mRNA degradation, and signal amplification, collectively reinforcing the spatial complexity of RNA silencing machinery in potato.

### 3.9. Evolutionary Ka/Ks ratio analysis of *StDCL*, *StAGO*, and *StRDR* in potato

**Fig 9:**
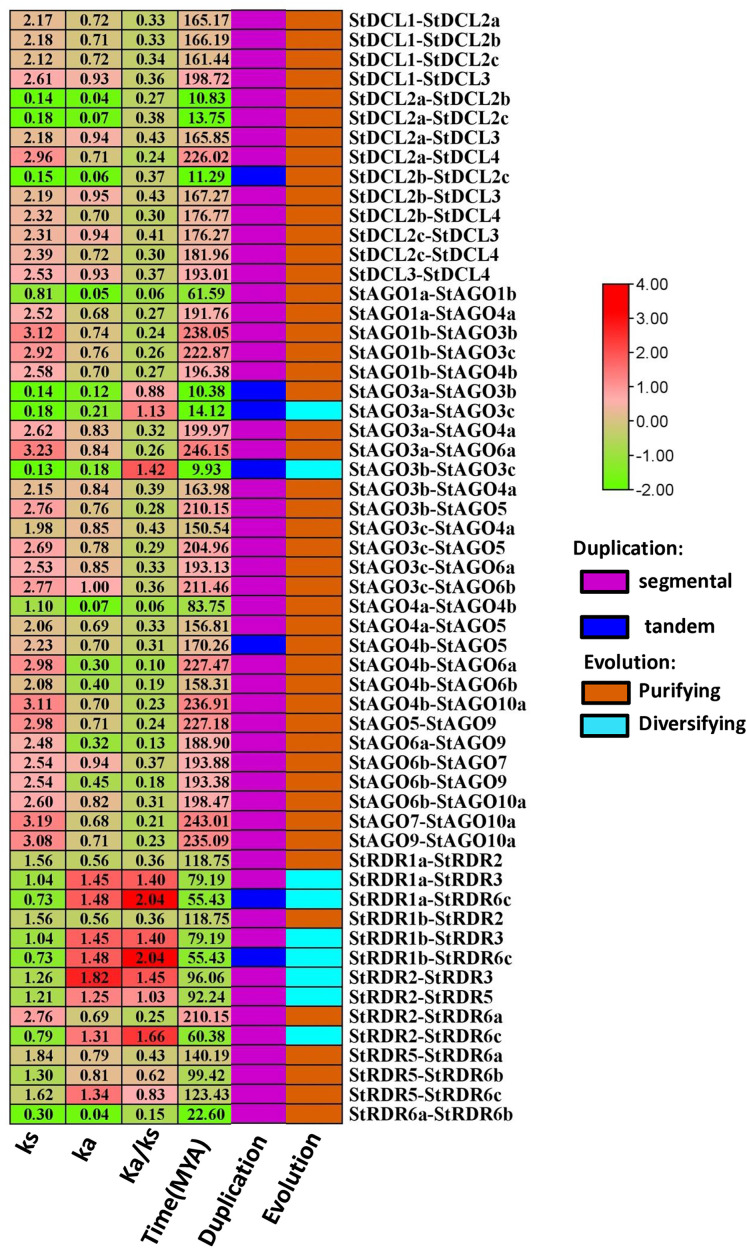
Estimation of gene duplications, evoluation, duplication time and Ka/Ks analysis of *StAGO*, *StRDR*, and *StDCL.* The ratio of Ka to Ks is represented by Ka/Ks, with divergence time (measured in MYA) also indicated. The color bar represents the data range. Visualization of Tandem duplications (dark blue) and segmental duplications (magenta) are mapped alongside purifying (orange) and diversifying (cyan) selection pressures.

The evolutionary dynamics of the *StDCL*, *StAGO*, and *StRDR* gene families were analyzed using the Ka/Ks ratio, which provides insights into the selective pressures acting on these genes (**Fig 9**). The Ka/Ks ratio measures the rate of non-synonymous (Ka) to synonymous (Ks) substitutions. We can identify purifying evolution, neutral evolution, and diversifying evolution from the ratio [118].

The Ka/Ks ratios for *StDCL* gene pairs ranged from 0.24 to 0.43. The lowest ratio was observed for *StDCL2a*-*StDCL4* (0.24), while the highest ratios were for *StDCL2a*-*StDCL3* and *StDCL2b*-*StDCL3* (0.43). All *StDCL* gene pairs are under purifying evolution, consistent with functional constraints expected for RNAi pathway genes then the *StAGO* family showed Ka/Ks ratios from 0.06 to 1.42. Most pairs, such as *StAGO1a*-*StAGO1b* (0.06) and *StAGO4a*-*StAGO4b* (0.06), exhibited purifying evolution. However, *StAGO3a*-*StAGO3c* (1.13) and *StAGO3b*-*StAGO4a* (1.42) showed diversifying evolution, suggesting adaptive evolution in response to environmental or pathogenic challenges. This diversification reflects their roles in gene regulation and viral defense. Similar evolutionary patterns have been reported in *Arabidopsis* and rice, where *AGO* and *DCL* diversification is often linked to adaptation against lineage-specific viruses and stress-responsive pathways [119]. Such selective pressures indicate that certain *AGO* members may have undergone functional divergence to fine-tune sRNA-mediated silencing under biotic stress [120]. The *StRDR* family displayed Ka/Ks ratios from 0.22 to 2.04. Pairs like *StRDR1a*-*StRDR2* (0.36) and *StRDR5*-*StRDR6a* (0.43) were under purifying evolution, while *StRDR1a*-*StRDR6c* (2.04) and *StRDR2*-*StRDR6c* (1.66) showed diversifying evolution. These adaptive changes may enhance RNA silencing efficiency against viral RNAs, whereas conserved pairs maintain core RNAi functions. Diversifying evolution was observed in *StAGO3a*-*StAGO3c*, *StAGO3b*-*StAGO3c*, *StRDR1a*-*StRDR3*, *StRDR1a*-*StRDR6c*, *StRDR1b*-*StRDR3*, *StRDR1b*-*StRDR6c*, *StRDR2*-*StRDR3*, *StRDR2*-*StRDR5*, and *StRDR2*-*StRDR6c* pairs, suggesting adaptive evolution in these gene pairs. In contrast, all remaining examined pairs exhibited purifying selection, indicating strong functional conservation.

We investigated most of the gene pairs are occurred segmental duplication. And the analysis revealed several gene pairs resulting from tandem duplication events, including *StDCL2b*-*StDCL2c* in the *StDCL* family, *StAGO3a*-*StAGO3b*, *StAGO3a*-*StAGO3c*, *StAGO3b*-*StAGO4a*, and *StAGO4b*-*StAGO5* in the *StAGO* family, as well as *StRDR1a*-*StRDR6c* and *StRDR1b*-*StRDR6c* in the *StRDR* family. These duplication events represent important evolutionary mechanisms that have contributed to the expansion and functional diversification of these gene silencing gene families in plants, often followed by either sub-functionalization or neo-functionalization under selective constraints [121,122]. These duplication-driven expansions provide genetic plasticity that may allow differential gene regulation, adaptation to diverse environmental conditions, and reinforcement of silencing efficiency under stress.

### 3.10. Synteny relationship analysis of *AGO*, *RDR*, and *DCL* gene families in potato and *Arabidopsis*

**Fig 10:**
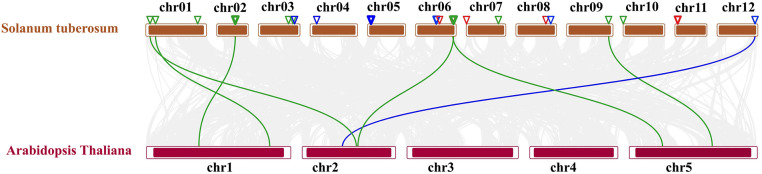
Syntenic relationships between *Solanum tuberosum* and *Arabidopsis thaliana* for RNAi genes.

To explore the evolutionary relationships and functional conservation of key gene families involved in RNAi pathways, synteny analysis was conducted between *Solanum tuberosum* and *Arabidopsis thaliana*. The study identified significant syntenic associations across the 12 chromosomes of *S. tuberosum* and the 5 chromosomes of *A. thaliana*, revealing conserved genomic regions and evolutionary patterns which are shown at **Fig 10**.

The *StAGO* gene family demonstrated strong syntenic conservation, with multiple homologous regions identified between *S. tuberosum* chromosomes Chromosome 01, Chromosome 02, Chromosome 06, and Chromosome 09 and *A. thaliana* chromosomes including Chromosome 1, Chromosome 2 and Chromosome 5. These conserved syntenic blocks suggest a shared evolutionary origin and functional conservation of *StAGO* genes, which are known to play critical roles in RNAi and gene regulation pathways. The preservation of these syntenic relationships underscores the importance of *AGO* genes in maintaining RNAi machinery across plant species. Similar *AGO* syntenic conservation has been previously observed among diverse angiosperms, reflecting the evolutionary stability of the RISC components across monocots and dicots [119,123].

Similarly, the *StRDR* gene family exhibited syntenic connections, primarily between *S. tuberosum* Chromosome 12 and *A. thaliana* Chromosome 2. This specific alignment highlights functional conservation of *StRDR* genes, which are essential for *RDR* activities. These enzymes are crucial for amplifying RNAi signals, contributing to antiviral defense and gene silencing mechanisms. The conserved positioning of *RDR* orthologs across species has been reported to correspond with stable transcriptional regulation and stress-responsive amplification of double-stranded RNA in higher plants [124,125]. The syntenic conservation of *StRDR* genes suggests that their roles in these pathways have been evolutionarily maintained.

The *StDCL* gene family did not show strong one-to-one syntenic alignment with A. thaliana chromosomes, though dispersed homologous regions were detected across several potato chromosomes, implying ancient segmental rearrangements and lineage-specific divergence [10,126].

The *StDCL* gene family not showed significant syntenic alignments, particularly between *S. tuberosum* Chromosome and *A. thaliana* but they are presented at various chromosome.

Overall, the synteny analysis reveals a high degree of evolutionary conservation among the RNAi gene families, emphasizing their critical roles in RNAi mechanisms like other studies [19]. The conservation of these RNA silencing components across taxonomic lineages reflects an evolutionary constraint imposed by their indispensable roles in post-transcriptional and transcriptional gene silencing. Such cross-species syntenic parallels strengthen the evidence that the molecular framework of RNAi is a deeply rooted regulatory system preserved throughout plant evolution [63,127]. These findings provide valuable insights into the evolutionary dynamics and functional conservation of RNAi-related genes in plants, highlighting the potential for cross-species functional studies to deepen our understanding of RNAi pathways.

### 3.11. PPI network analysis of RNAi-related genes in potato

**Fig 11:**
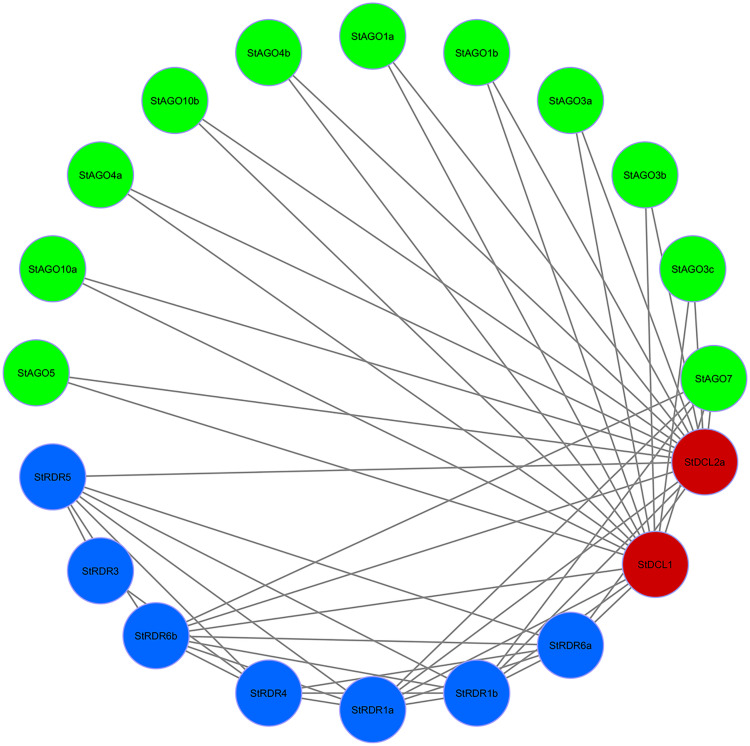
PPI network of RNAi-related genes in potato, where red color, blue color and green color indicate *StDCL*, *StRDR* and *StAGO* respectively.

The PPI analysis sought to explore the relationships of 29 RNAi-associated genes identified in potato. PPI network analysis visualized 20 genes (**Fig 11**) showcasing intricate reciprocal relations among *RDR*, *AGO*, and *DCL* gene families.

In the PPI network, stronger interconnectivity was shown between *StDCL1* and *StDCL2a* with several *StRDR* and *StAGO* genes, which may point toward their importance in silencing cascades [128]. *StDCL1* specifically interacted with 15 genes, including *StRDR6a*, *StRDR6b*, *StRDR1a*, *StRDR1b*, as well as several members of the *StAGO* family (*StAGO1a*, *StAGO1b*, *StAGO3a*, *StAGO3b*, *StAGO3c*, *StAGO4a*, *StAGO4b*, *StAGO10a*, *StAGO10b*, and *StAGO7*).

*StDCL2a* was highly interconnected in a similar manner, interacting with 15 genes, including *StRDR1a*, *StRDR1b*, *StRDR6a*, *StRDR6b*, and various *StAGO* proteins (*StAGO1a*, *StAGO1b*, *StAGO3a*, *StAGO3b*, *StAGO3c*, *StAGO4a*, *StAGO4b*, *StAGO10a*, *StAGO10b*, and *StAGO7*).

Besides, *StAGO7* was found to interact with *StRDR1a*, *StRDR1b*, and *StRDR6b*, furthering its role in bi-directionally bridging *AGO* and *RDR* proteins. Notably, *StRDR1a* and *StRDR1b* were found interacting together and with several other partners like *StRDR6a*, *StRDR6b*, *StDCL1*, *StDCL2a*, and *StAGO7*, implying their functional redundancies or possible bulk participation in the silencing pathway [129]. But beyond the core interactions, the PPI network shows *StRDR4* to be a highly connected node, interacting with *StRDR1a*, *StRDR1b*, *StRDR3*, *StRDR5*, *StRDR6a*, and *StRDR6b*, which again suggests a regulatory or bridging role within the *RDR* family [130].

StRDR5 implicated in networks with *StDCL2a*, *StRDR1a*, *StRDR1b*, *StRDR6a*, and *StRDR6b* fosters further cross-talk within the *RDR* family and with *DCL* proteins. Unlike other *AGOs*, *StAGO5* formed connections only with *StDCL1* and *StDCL2a*, hinting at a specialization in linking *AGO* with *DCL* functions [131].

The extensive interactions among *DCL*, *AGO*, and *RDR* genes discovered are a reflection of the conserved RNAi mechanism where these proteins are involved in sRNA processing and gene silencing activities. The high connecting power of *StDCL1*, *StDCL2a*, *StRDR1a*, *StRDR1b*, and *StAGO7* also signifies the key roles these components play in the network and perhaps as hubs for coordinating silencing efficiency [132,133].

These insights agree with the classical RNAi pathway models, where dsRNA is processed by *DCL* into siRNAs that are loaded into *AGO* proteins to target cleavage of complementary RNAs, while *RDR* provides enhancement for the silencing pathway by creating secondary siRNAs. The observed interactions point to a fine-regulated and cooperative Potato system, thereby adding to the evolutionarily conserved nature of these mechanisms [134,135].

### 3.12. GO analysis of *StAGO*, *StDCL*, and *StRDR* in *Solanum tuberosum*

GO analysis underscored the conserved roles of RNAi genes in antiviral defense and RNA silencing while highlighting their dynamic regulatory interplay in plant immunity, as evidenced by immune-related GO enrichment studies in other systems [136,137]. This reinforces their functional significance in *Solanum tuberosum*, where their characterization could unravel novel mechanisms of pathogen resistance and intracellular coordination.

**Fig 12:**
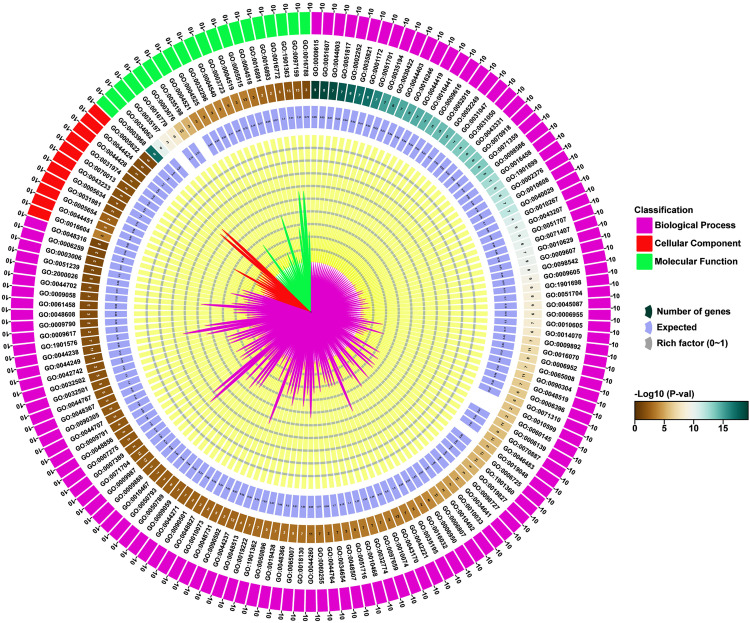
*StAGO*, *StDCL*, and *StRDR* gene functions were analyzed by GO and visualized on the right of the circos plot, where gene counts per GO term, expected values, and rich factors are color-coded, and −log₁₀(p-value) is shown with three distinct colors.

The GO analysis identified 157 annotations across three categories: biological processes (BP), cellular components (CC), and molecular functions (MF) shown at **Fig 12** and **S4 File**. Biological processes dominated with 126 terms (p-values: 5.4 × 10 ⁻ ^20^ to 0.04789), while cellular components and molecular functions comprised 11 (p-value range: 0.00021–0.03323) and 20 terms (p-value range: 8.4 × 10 ⁻ ^18^–0.0344), respectively.

Here we also identified several key terms associated with gene silencing and RNAi, including posttranscriptional gene silencing by RNA (GO: 0035194), RNAi (GO: 0016246), and production of siRNA (GO: 0030422), highlighting the central role of RNAi in regulating gene expression. Additional terms such as virus-induced gene silencing (GO: 0009616) and gene silencing by miRNA (GO: 0035195) underscore the involvement of RNAi in antiviral defense and miRNA-mediated regulation. Molecular functions like RNA-directed RNA polymerase activity (GO: 0003968) and siRNA/miRNA binding (GO: 0035197, GO: 0035198) further emphasize the enzymatic and effector mechanisms of RNAi, involving proteins such as *DCLs* (*StDCL1*, *StDCL2a*), *RDRs* (*StRDR6b*, *StRDR1b*), and *AGOs* (*StAGO1a*, *StAGO4a*). These findings collectively illustrate the diverse roles of RNAi in gene regulation, stress response, and pathogen defense. Nucleic acid binding and protein binding (13 genes each) underscored critical interactions for RISC formation. *StDCLs* protein exhibited ribonuclease III activity, processing dsRNA into siRNAs, while *StAGOs* bound siRNAs/miRNAs for sequence-specific silencing. These conserved mechanisms underpin RNAi roles in antiviral defense, development, and stress responses [138,139].

The functional divergence of RNAi gene families reflects specialized roles in potato antiviral defense, as supported by studies integrating RNA silencing and CRISPR/Cas systems for engineered resistance [140,141]. Moreover, the enrichment of antiviral and silencing-related GO terms suggests that these gene families operate as central nodes in the RNA-based immune network, coordinating transcriptional and post-transcriptional repression during pathogen attack [142,143]. Such molecular crosstalk between sRNA pathways and stress-signaling cascades is increasingly recognized as a conserved defense paradigm across angiosperms, ensuring robust adaptation under viral and abiotic stress conditions [65,144]. These GO insights not only reinforce the evolutionary conservation of RNAi machinery but also highlight its potential exploitation for precision genome engineering and development of durable virus-resistant cultivars through combined RNAi–CRISPR strategies [145,146].

### 3.13. Collinearity relationship analysis of *StAGO*, *StDCL*, and *StRDR* in *Solanum tuberosum*

**Fig 13:**
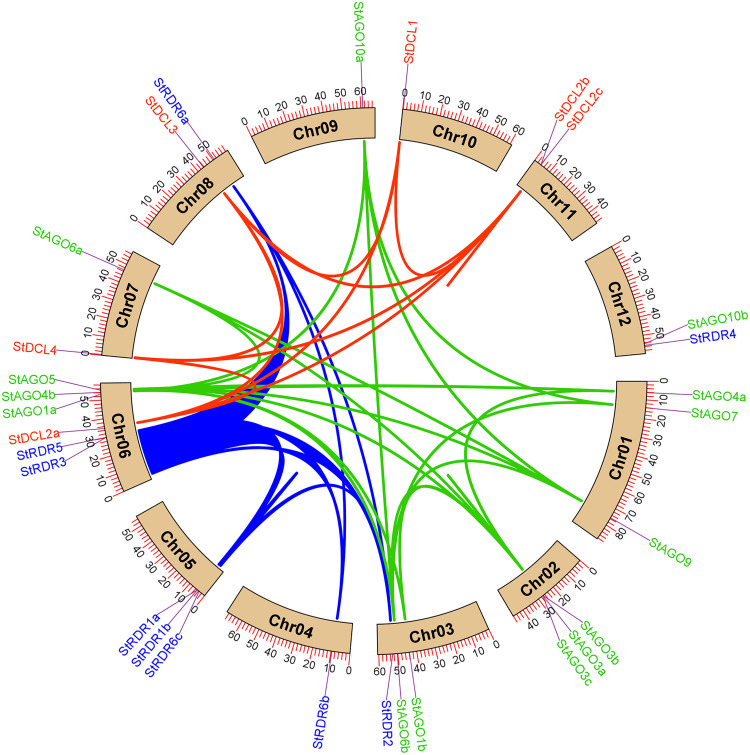
Collinearity relationships among *StAGO*, *StRDR*, and *StDCL* gene families.

Collinearity analysis revealed 57 connections among *StAGO*, *StDCL*, and *StRDR* gene families in potato, elucidating their evolutionary and functional relationships which shown at **Fig 13**. These connections reflect duplication and divergence events shaping RNA silencing pathways [147,148].

*StDCL* family exhibited 14 collinear connections, including *StDCL1* (chromosome 10) linked to *StDCL2a* (chromosome 6), *StDCL2b/StDCL2c* (chromosome 11), and *StDCL3* (chromosome 8). *StDCL2a* further interacted with *StDCL3* and *StDCL4* (chromosome 4), suggesting segmental duplications driving functional diversification [147]. *StAGO* family showed the highest collinearity (29 connections), indicating complex evolutionary dynamics. Key interactions included *StAGO1a* (chromosome 5) with *StAGO1b* (chromosome 5) and *StAGO4a* (chromosome 1), and *StAGO4b* (chromosome 6) with *StAGO5* (chromosome 6) and *StAGO10a* (chromosome 9). These patterns suggest sub-functionalization of *AGO* members in sRNA-mediated regulation [148]. *StRDR* family displayed 14 connections, such as *StRDR1a* (chromosome 6) pairing with *StRDR2* (chromosome 3) and *StRDR6c* (chromosome 5). *StRDR2* linked to *StRDR5* (chromosome 6) and *StRDR6a* (chromosome 8), reflecting conserved roles in dsRNA amplification and silencing enhancement [147]. The extensive collinearity among these RNAi gene families suggests that gene duplication and chromosomal rearrangements have contributed to the diversification of silencing pathways in potato. This genomic organization provides evolutionary flexibility, allowing functional specialization of *AGO*, *DCL*, and *RDR* proteins for distinct silencing processes such as antiviral RNAi, transposon suppression, and epigenetic regulation as like other Plants such as Rice, Maize [119,149]. Moreover, the co-localization of certain gene pairs across chromosomes may facilitate coordinated expression during pathogen attack or developmental transitions, strengthening the integrated silencing network in potato [150]. Similar collinearity-driven expansion of RNAi machinery has been reported in other plants such as *Arabidopsis thaliana* and *Oryza sativa*, emphasizing the evolutionary conservation of RNA silencing as a genomic defense system [151,152].

### 3.14. CREs analysis in the promoters of *StDCL*, *StAGO*, and *StRDR*

**Fig 14:**
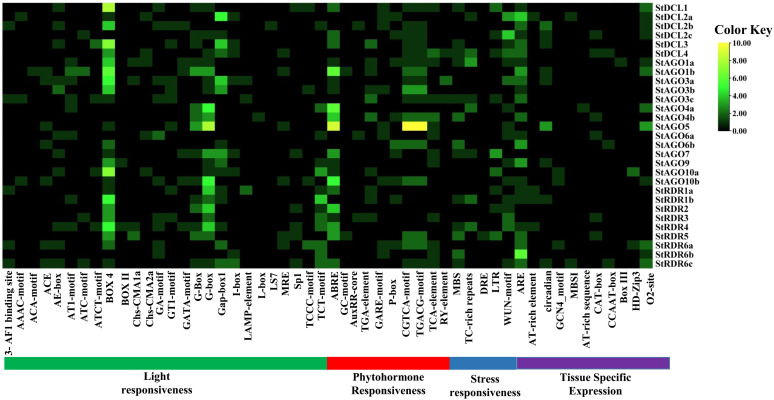
A heatmap represents the distribution of putative CAREs on the 2.0 kb promoter region of *StDCLs*, *StAGOs* and *StRDRs.* The names of the *StDCLs*, *StAGOs* and *StRDRs* genes are shown on the right side of the heat map. Functions associated with CAREs of the corresponding genes, such as light responsiveness (green), phytohormone responsiveness (red), stress responsiveness (blue), and tissue-specific expression (magenta) are indicated at the bottom.

CREs refer to non-coding DNA sequences that include short motifs typically ranging from 5 to 20 base pairs. These motifs act as binding sites for TFs, allowing them to attach to specific target genes to initiate transcription and regulate gene expression [153,154]. The advancement of high-throughput genome sequencing technology has significantly increased the availability of sequencing data for commercially important crops every year [155]. Consequently, bioinformatics tools can be utilized to efficiently explore databases and identify functional regulatory regions within DNA sequences—most commonly in promoter and enhancer regions—linked to particular gene functions. The analysis of CREs in the promoters of *StDCL*, *StAGO*, and *StRDR* revealed 52 elements, with the most abundant category being Box 4, related to light responsiveness. CAREs were classified into four categories based on their functional regulation: light responsiveness, tissue-specific expression, phytohormone responsiveness, and stress responsiveness (**Fig 14** and **S5 File**). The light response occurring in the leaf tissue of potato plants is significantly affected by photosynthesis, an essential physiological process [156].

The largest category of CAREs related to light responsiveness included 26 elements, such as 3-AF1 binding site, AAAC-motif, ACA-motif, ACE, AE-box, AT1-motif, ATC-motif, ATCT-motif, Box 4, Box II, Chs-CMA1a, Chs-CMA2a, GA-motif, GT1-motif, GATA-motif, G-Box, G-box, Gap-box1, I-box, LAMP-element, L-box, LS7, MRE, Sp1, TCCC-motif, and TCT-motif. The phytohormone responsiveness CAREs included 10 elements, such as ABRE, GC-motif, AuxRR-core, TGA-element, GARE-motif, P-box, CGTCA-motif, TGACG-motif, TCA-element, and RY-element. The stress responsiveness category included five elements, such as MBS, TC-rich repeats, DRE, LTR, and WUN-motif. The tissue specific expression category included 11 elements, such as ARE, AT-rich element, circadian, GCN4_motif, MBSI, AT-rich sequence, CAT-box, CCAAT-box, Box III, HD-Zip3, and O2-site. This comprehensive analysis highlights the diverse regulatory mechanisms governing the expression of *StDCL*, *StAGO*, and *StRDR*, emphasizing their roles in light signaling, tissue-specific functions, phytohormone responses, and stress adaptation.

In this analysis, the light-responsive elements Box 4 and G-box demonstrated the highest frequency of occurrence across multiple genes. Among tissue-specific elements, ABRE showed particularly widespread distribution, appearing in numerous gene promoters, while CGTCA-motif and TGACG-motif were also prominently represented. Within the phytohormone-responsive category, these same elements (CGTCA-motif and TGACG-motif) again displayed significant prevalence. Regarding stress-responsive elements, ARE and O2-site emerged as the most abundant, appearing frequently across various gene families in all analyzed promoter sequences.

The presence of these regulatory motifs in RNAi-associated gene promoters suggests an intricate transcriptional control network that links environmental cues to PTGS. CAREs such as ABRE and MBS are known to mediate abscisic acid and drought-responsive transcription, which can activate *RDR* and *DCL* expression during stress, enhancing sRNA biogenesis [157,158]. Similarly, light-responsive motifs (G-box, GT1-motif) may synchronize *AGO* and *DCL* gene activity with diurnal or developmental regulation, maintaining the stability of sRNA populations under varying light conditions [159]. The enrichment of stress- and hormone-responsive elements reflects the adaptive flexibility of RNAi genes in modulating defense signaling cascades under pathogen attack, consistent with the transcriptional upregulation of *AGO* and *RDR* members observed during viral infection and abiotic stress in other plants [65,160].

These findings collectively highlight that cis-element diversity contributes to the spatiotemporal expression of silencing genes in potato, integrating transcriptional and post-transcriptional layers of regulation to ensure robust RNA silencing and stress tolerance mechanisms.

### 3.15. Regulatory network between TFs and *StDCL*, *StAGO*, and *StRDR* in *Solanum tuberosum*

**Fig 15:**
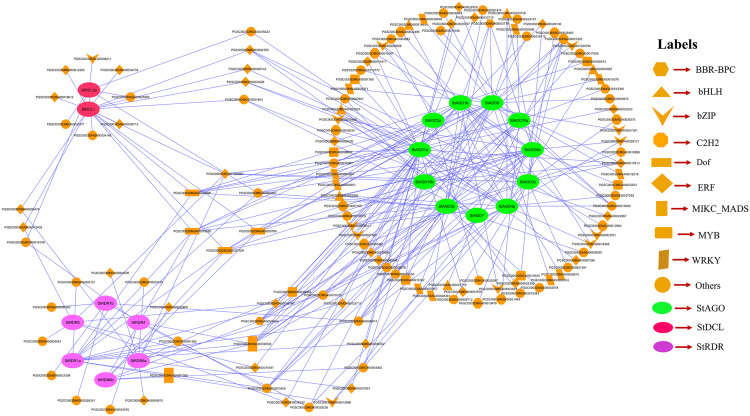
The regulatory network among the TFs and the predicted RNAi genes. The *StDCL*, *StAGO*, and *StRDR* genes were represented by red, green, and pink node color, respectively, and the TFs were represented by yellow node color with different shape for different families of TFs.

A comprehensive analysis of TFs regulating *StAGOs*, *StDCLs*, and *StRDRs* in *Solanum tuberosum* identified a total of 132 unique TFs, categorized into 25 distinct families (**S6 File****).** Notably, TFs from major families such as ERF, Dof, MYB, bZIP, BRB-BPC, and MIKC-MADS play pivotal roles in regulating these gene groups which are shown at **Fig 15**.

In the *StDCL*–*StAGO*–*StRDR* interaction set, five TFs establish connections across all three gene families. These include PGSC0003DMG401023951 (ERF family), PGSC0003DMG400019528 (Dof family), PGSC0003DMG400002507 (ERF family), PGSC0003DMG400000786 (Dof family), and PGSC0003DMG400037029 (Dof family). Additionally, PGSC0003DMG400018190 interacts within this set, though it belongs to a TF group outside the primary families considered.

Within the *StDCL*–*StAGO* interaction set, three TFs PGSC0003DMG400002350, PGSC0003DMG400009142, and PGSC0003DMG400030228 are identified as members of the ERF family, underscoring the prominent role of ERF TFs in regulating both *DCL* and *AGO* genes. Other TFs, such as PGSC0003DMG400015424 and PGSC0003DMG400004953, also show interactions but are classified into other families not central to this analysis.

In the *StDCL*–*StRDR* interaction set, PGSC0003DMG400009475 and PGSC0003DMG400013402, both from the ERF family, interact across these gene classes. Another TF, PGSC0003DMG400016148, is also involved but is associated with other less defined TF groups.

The *StAGO*–*StRDR* interaction set exhibits extensive regulatory connections involving multiple TFs from diverse families. These include PGSC0003DMG400004948 (MYB family), PGSC0003DMG400010075 and PGSC0003DMG400023426 (Dof family), PGSC0003DMG400002185 and PGSC0003DMG400014541 (ERF family), PGSC0003DMG402029444 (BRB-BPC family), PGSC0003DMG400000008 (MIKC-MADS family), PGSC0003DMG400004062 (Dof family), and PGSC0003DMG400007951 and PGSC0003DMG400026232 (ERF family). PGSC0003DMG400012660, originally classified in the bZIP family, also establishes connections within this group. Additional TFs like PGSC0003DMG400623717 and PGSC0003DMG400009914 are noted after sequence formatting corrections but require further validation.

**Fig 16:**
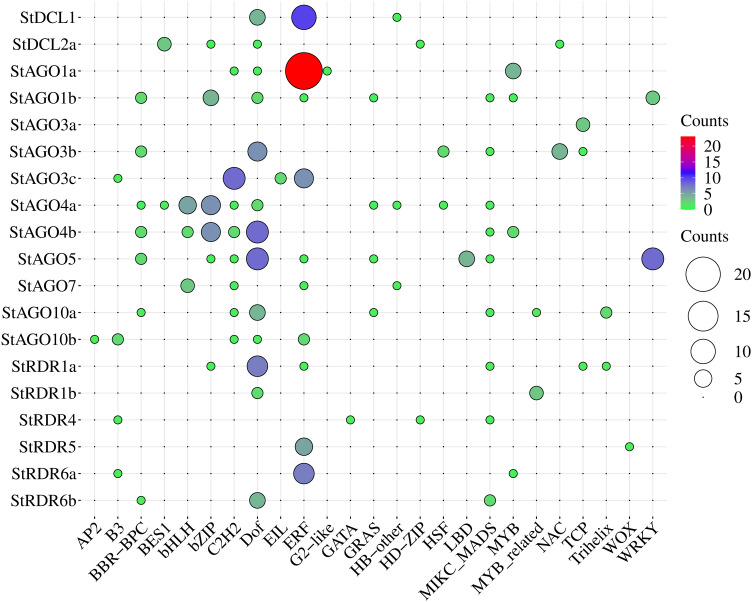
Balloon plot represents the related number of TFs with the predicted StRNAi genes with *StDCL*, *StAGO*, and *StRDR* proteins on the left and TF names at the bottom. Color intensity and size indicates number of TF presence for each proteins.

Balloon plot analysis indicates that *StAGO1A* has the highest interaction frequency with the ERF family, with 20 interactions recorded, which shown at **Fig 16**. Strong interactions are also observed between *StDCL1* and members of the ERF family, and between *StAGO3c*, *StRDR6a*, and *StRDR5* with the ERF family, where *StRDR6A* exhibits a stronger interaction profile compared to *StRDR5* and *StRDR3C*. *StAGO3C* additionally forms significant interactions with the C2H2 family, while *StAGO4b*, *StAGO5*, and *StRDR1a* are connected to the Dof family. *StAGO5* further establishes links with the WRKY family. Moderate interaction levels (10–15 counts) are observed for TF families such as DOF, ERF, bHLH, and bZIP, with lower-level interactions occurring with MYB, NAC, and MIKC-MADS TFs across the *DCL*, *AGO*, and *RDR* gene members.

**Fig 17:**
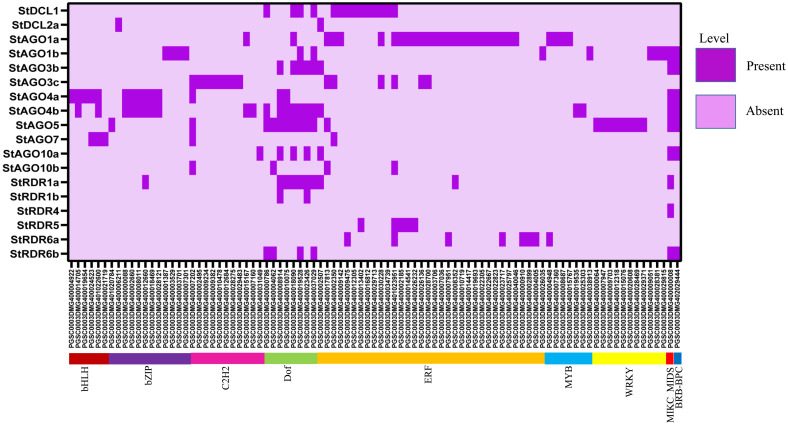
A heatmap depicts TF families regulating potato RNAi-related genes, with *StDCL*, *StAGO*, and *StRDR* proteins on the left and TF names at the bottom. Color intensity indicates TF presence for each protein.

A comprehensive analysis of TFs regulating *StAGOs*, *StDCLs*, and *StRDRs* in *Solanum tuberosum* identified the TFs bHLH, bZIP, C2H2, Dof, ERF, MYB, WRKY, MIKC_MADS, and BRB-BPC families may play significant role in regulating RNAi genes (**Fig 17**). Specifically, only seven TF families (ERF, bZIP, C2H2, WRKY, Dof, MYB and bHLH) are connected 67.42% out of total connections. Additionally we also investigated that the single PGSC0003DMG402029444 (BBR-BPC) and PGSC0003DMG400000008 (MIKC_MADS) are connected with more than five RNAi genes. The ERF family emerged as the most prominent, comprising 34 TFs, followed by the bZIP family with 12 TFs. The C2H2 and WRKY families each included 11 TFs, while the Dof and MYB families consisted of 8 and 7 TFs, respectively. The bHLH family contributed 6 TFs, and the MIKC_MIDS and BRB-BPC families were represented by a single TF each. This diverse array of TFs highlights the complexity of the regulatory network governing these genes in potato.

These findings align with previous studies highlighting the roles of ERF [161], Dof [162], MYB [163], and bZIP [164] TF families in plant development and stress responses. Specifically, the ERF family is known for its involvement in various stress responses in potatoes [161], while the bZIP family has been implicated in gene diversification and spatiotemporal gene expression in *Solanum tuberosum* [165]. The MYB family also plays multiple roles in development and stress responses in potatoes [163].

The prominent enrichment of ERF, Dof, and MYB TFs within RNAi-associated networks underscores a regulatory bridge between transcriptional control and post-transcriptional silencing. ERF and WRKY TFs, for instance, are known to modulate RNA silencing genes under viral or abiotic stress, coordinating defense-related expression of AGO and RDR members [166,167]. Likewise, Dof and bZIP TFs are implicated in hormonal signaling and transcriptional reprogramming during pathogen challenge, which can activate or repress RNAi gene modules [164]. These multilayered connections reflect an integrated feedback loop where TFs modulate sRNA machinery to fine-tune gene silencing, thereby contributing to adaptive stress responses and genome defense in potato [163,168]. Such regulatory coupling may represent an evolutionary optimization that ensures transcriptional flexibility and RNAi robustness under environmental pressures.

### 3.17. Prediction of potential miRNAs targeting *StDCL*, *StAGO* and *StRDR* genes in *Solanum tuberosum*

**Fig 18:**
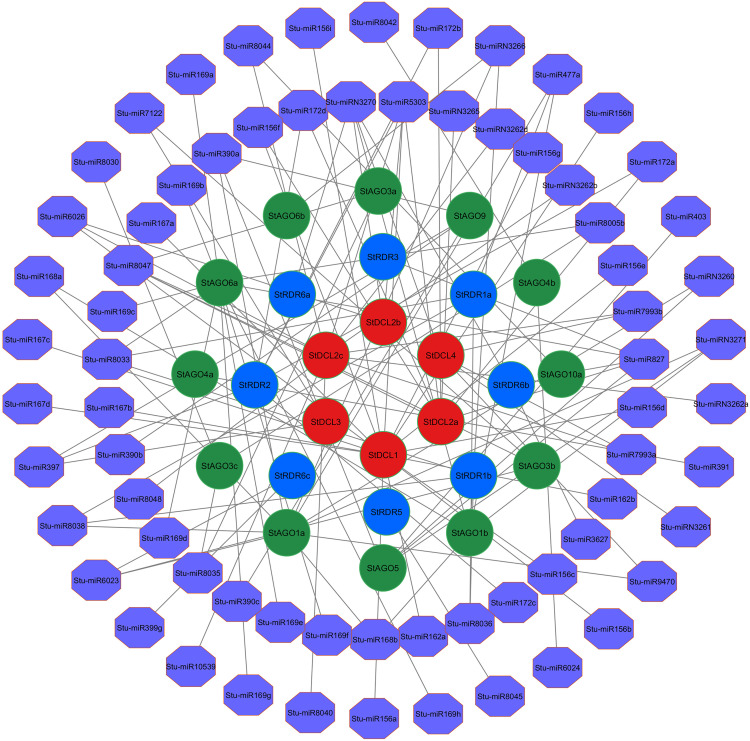
miRNAs interaction network of RNAi-related genes in potato, where purple color, red color, blue color and green color indicate miRNAs, *StDCL*, *StRDR* and *StAGO* respectively.

In this analysis, a total of 117 miRNA-target interactions were identified, affecting 26 genes from the *StDCL*, StAGO, and *StRDR* families. The investigation uncovered 68 unique miRNA sequences which are shown at **Fig 18**, with stu-miR827, stu-miR5303 and stu-miRN3270 being the most prevalent, each appearing five times. Among the targeted genes, *StDCL1* (17), *StDCL2a* (5), *StDCL2c* (7), *StAGO1a* (6), *StAGO1b* (5), *StRDR1a* (2), *StRDR1b* (2), and *StRDR2* (5) were the most frequently targeted.

The findings indicate that stu-miR827 targets several genes, including *StAGO5*, *StRDR3*, *StAGO6a*, *StDCL2a*, and *StAGO10a*. This miRNA is not only evolutionarily conserved but also associated with phosphate starvation responses in plants, consistent with prior studies linking miR827 to nutrient stress regulation and homeostasis [169]. Its multiple interactions with *AGO* and *RDR* genes imply a role in adjusting RNA silencing pathways during nutrient stress, which may help balance growth and adapt to stressful conditions [90].

In contrast, stu-miR5303 targets genes are *StDCL2c*, *StDCL1*, *StDCL2b*, *StDCL2a*, and *StRDR2* and the miRNA has previously been identified in Various Plants miRNA family [170], suggesting a lineage-specific adaptation mechanism. It is thought to regulate isoform-specific *DCL* activity, thereby fine-tuning miRNA biogenesis and reinforcing the dynamic feedback between miRNA productions and silencing control [171]. Notably, the presence of *StRDR2* indicates potential crosstalk between miRNA and siRNA pathways, highlighting the cooperative regulation within the RNAi machinery..

Lastly, stu-miRN3270 interacts with *StAGO1b*, *StDCL4*, *StDCL2b*, *StRDR6b*, and *StRDR6a* and its targeting of StRDR6—a key amplifier of secondary siRNA production—suggests a conserved mechanism of miRNA-mediated feedback in PTGS [172]. This interaction pattern implies that miRN3270 could influence systemic silencing and antiviral defense, consistent with previous evidence that *RDR6*-associated miRNAs enhance long-distance silencing and viral resistance [173].

Overall, these findings reveal the intricate regulatory interplay between miRNAs and RNA silencing components in potato, providing insights into how sRNAs orchestrate stress adaptation and antiviral defense in crop plants and the highly connected miRNA genes are summarized in **Table 3**.

**Table 3:** Information of highly connected potential miRNAs targeting *StDCL*, *StAGO*, and *StRDR* genes in *Solanum tuberosum.*

miRNA	Occurrences	Targets
Stu-miR827	5	*StAGO5; StRDR3; StAGO6a; StDCL2a; StAGO10a*
Stu-miR5303	5	*StDCL2c; StDCL1; StDCL2b; StDCL2a; StRDR2*
Stu-miRN3270	5	*StAGO1b; StDCL4; StDCL2b; StRDR6b; StRDR6a*
Stu-miR156f	4	*StDCL2a; StDCL1; StDCL1; StDCL1*
Stu-miR168a	4	*StAGO1a; StAGO1b; StAGO1a; StAGO1b*
Stu-miR168b	4	*StAGO1a; StAGO1b; StAGO1a; StAGO1b*
Stu-miR172a	4	*StRDR2; StRDR2; StDCL2a; StDCL2a*
Stu-miR397	4	*StAGO9; StRDR2; StDCL3; StAGO9*

### 3.18. Comprehensive gene expression profiling across tissues, developmental stages, and stress conditions of RNAi-related genes

**Fig 19:**
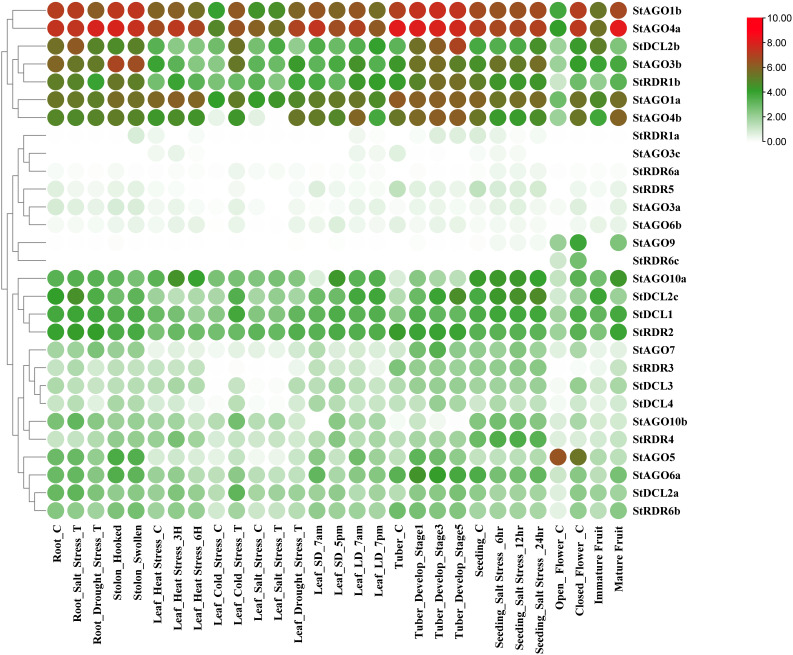
Comprehensive gene expression pattern of *StDCL*, *StRDR* and *StAGO* across tissues, developmental stages, and stress conditions are represented at the bottom of the heat map. The respective *StDCL*, *StRDR* and *StAGO* gene names are shown on the right side of the heat map. The color gradient from white-green-red indicates the expression levels on the right side of the heat map.

The tissue-specific expression analysis of RNAi-associated genes revealed variable expression patterns across potato tissues, developmental stages and stress conditions which shown at **Fig 19**. In root tissues, *StAGO1b*, *StAGO3b*, *StAGO4a*, and *StDCL2b* were highly expressed under both control and stress conditions, particularly under salt and drought stress. Expression of *StAGO3b* remained consistently high in all root samples, suggesting a prominent role in root development and stress response. These findings align with previous studies indicating that genes such as *StRD22* and *StERD7* are upregulated under drought conditions in potato roots, contributing to stress tolerance mechanisms [162,174]. Such expression dynamics further reinforce the central role of RNAi machinery in modulating abiotic stress responses through post-transcriptional regulation [65].

In stolon tissues, especially during the hooked and swollen stages, *StAGO4a* and *StAGO3b* showed strong expression. These genes were also highly expressed in the stolon tip, suggesting involvement in early tuber formation. In contrast, *StDCL2b*, *StRDR1b*, and *StRDR6b* displayed lower or no expression in these tissues. Similar tissue-specific expression patterns have been observed in the *StSOS1* gene family, where certain members are predominantly expressed in stolon tissues, indicating their role in tuber development [175]. This parallel expression between silencing-related genes and developmental regulators highlights how sRNA pathways coordinate hormonal and developmental signaling during tuber initiation [176].

Leaf tissues exhibited relatively uniform expression profiles. *StAGO1b*, *StAGO4a*, and *StAGO6* showed higher expression levels under heat stress (3 h and 6 h), while cold-stressed leaves displayed reduced expression for most RNAi genes. *StAGO4a* and *StDCL3a* were among the few genes with moderate expression under cold conditions. This pattern is consistent with the expression of stress-responsive genes such as *StEREBP1*, which is upregulated under various environmental stresses, including low temperature, enhancing tolerance in transgenic potato plants [177]. The modulation of *AGO* and *DCL* members under temperature extremes supports the hypothesis that RNA silencing components fine-tune stress gene expression through temperature-sensitive regulatory loops [178].

In petiole tissue, *StAGO1b*, *StAGO4a*, and *StAGO6* were actively expressed, along with moderate expression of *StDCL2b* and *StDCL3a*. Expression patterns in stem and shoot apex were similar, with *StAGO1b*, *StAGO4a*, and *StAGO6* showing consistently higher expression. These observations are in line with studies demonstrating that genes involved in hormone signaling pathways exhibit tissue-specific expression, contributing to plant development and stress responses [19]. The consistent presence of *AGO1b* and *AGO4a* across vegetative organs indicates their likely housekeeping function in maintaining endogenous sRNA balance for growth regulation [179].

In reproductive organs, including seeding, flower, and fruit tissues, several genes showed stage-specific expression. *StAGO1b*, *StAGO4a*, and *StDCL3a* exhibited strong expression in flower bud, closed flower, and mature flower tissues. In fruit tissues, *StAGO1b* and *StAGO4a* were highly expressed in mature fruit, while *StAGO6* showed moderate expression in both young and mature stages. These findings suggest that RNAi-related genes may play roles in reproductive development, as supported by previous research on gene expression during microtuberization under various stress conditions [175]. Such temporal expression of silencing-related genes during reproductive organ formation suggests a conserved mechanism by which miRNA and siRNA pathways orchestrate developmental phase transitions [180].

In tuber-related tissues, expression levels varied with developmental stage. *StAGO1b*, *StAGO4a*, and *StDCL2b* showed increased expression during tuber development, particularly in later stages (Stage 3 and Stage 5). Expression was also high in sprouting tubers and in the tuber cortex. These genes also displayed similar expression patterns in multiple tuber-associated tissues, including peel, pith, and young tuber. The involvement of RNAi-related genes in tuber development and stress adaptation is further supported by studies highlighting the role of genes like *StProDH1* in enhancing drought tolerance through gene silencing approaches [181]. Such temporal expression of silencing-related genes during reproductive organ formation suggests a conserved mechanism by which miRNA and siRNA pathways orchestrate developmental phase transitions [180].

Overall, *StAGO1b*, *StAGO4a*, and *StAGO3b* were among the most widely expressed genes across all tissues, indicating broad functional roles in development and stress adaptation. Collectively, these expression profiles underscore the integrative role of RNAi-related genes as transcriptional and post-transcriptional regulators linking developmental plasticity, hormonal control, and stress resilience in potato [65,176,178–180].

## 5. Conclusion

In this study, we conducted the first genome-wide characterization of RNAi components in potato, identifying 6 *StDCL*, 14 *StAGO*, and 9 *StRDR* genes that mediate crucial gene regulation processes. Phylogenetic analysis with *Arabidopsis thaliana* orthologs confirmed their classification into established clades, while conserved domain and motif architectures underscored their functional integrity. Our comparative genomic analysis revealed strong syntenic conservation with *Arabidopsis*, highlighting evolutionary preservation of RNAi pathways. Furthermore, evolutionary rate (Ka/Ks) analysis indicated that the majority of duplicated gene pairs have undergone purifying selection, with divergence times estimated for key duplication events, providing insight into the evolutionary history of this gene family. PPI networks demonstrated extensive functional connectivity among these components, while GO analysis linked them to gene silencing and antiviral defense mechanisms. Subcellular localization predictions revealed a predominant nuclear and cytoplasmic presence for these proteins, aligning with their roles in transcriptional and PTGS. Regulatory network and sub-network analysis were identified important TFs; ERF, Dof, MYB, bZIP, BRB-BPC, and MIKC-MADS families, which are associated with *StDCL*, *StAGO*, and *StRDR* genes. We were also identified important mirnas; stu-miR827, stu-miR5303 and stu-miRN3270 which are associated with *StDCL*, *StAGO*, and *StRDR* genes. Examination of CREs identified abundant light-responsive (Box 4, G-box), tissue-specific (ABRE, CGTCA-motif, TGACG-motif), and stress-responsive (ARE, O2-site) motifs in their promoter regions. Gene structure analysis revealed a diversity of intron-exon patterns, with closely related phylogenetic clades often sharing similar architectural features. Notably, expression profiling showed *StAGO1b*, *StAGO4a*, and *StDCL2b* were highly expressed in roots, stolons and tubers, suggesting specialized roles in drought adaptation. These findings establish a molecular framework for potato RNA silencing research, enabling future applications in crop improvement through functional validation, disease resistance studies, and RNAi-assisted breeding strategies to enhance stress resilience and agricultural productivity.

## Supporting information

S1 File Full-length protein sequences of *DCL* gene families of *A. thaliana* and *S. tuberosum* plant species. (TXT)

S2 File Full-length protein sequences of *AGO* gene families of *A. thaliana* and *S. tuberosum* plant species. (TXT)

S3 File Full-length protein sequences of *RDR* gene families of *A. thaliana* and *S. tuberosum* plant species. (TXT)

S4 File The details GO analysis of the predicted RNAi related genes was performed using online tool of Plant Transcription Factor Database (PlantTFDB, http://planttfdb.cbi.pku.edu.cn//). (XLSX)

S5 File The predicted CREs of the upstream promoter region (2.0 kb genomic sequences) of RNAi gene families in potato. (XLSX)

S6 File Identified in total 240 TFs associated the regulation of predicted RNAi silencing genes in banana genome. (XLSX)

## Abbreviations

sRNAs: Small RNAs
miRNAs: MicroRNAs
siRNAs: Small Interfering RNAs
*DCL*: Dicer-like Protein
*AGO*: Argonaute
*RDR*: RNA-dependent RNA Polymerases
*RNAi*: RNA Interference
dsRNA: Double-stranded RNA
ssRNA: Single-stranded RNA
RdRP: RNA-dependent RNA Polymerase
*StDCL*: *Solanum tuberosum* Dicer-like
*StAGO*: *Solanum tuberosum* Argonaute
*StRDR*: *Solanum tuberosum* RNA-dependent RNA Polymerase
HMM: Hidden Markov Model
BLASTP: Basic Local Alignment Search Tool (Protein)
MEGA: Molecular Evolutionary Genetics Analysis
ML: Maximum Likelihood
MEME: Multiple Expectation Maximization for Motif Elicitation
GSDS: Gene Structure Display Server
MCScanX: Multiple Collinearity Scan toolkit
PPI: Protein-Protein Interaction
STRING: Search Tool for the Retrieval of Interacting Genes/Proteins
GO: Gene Ontology
CREs: Cis-Regulatory Elements
TFs: Transcription Factors
FPKM: Fragments Per Kilobase of transcript per Million mapped reads
Ka/Ks: Non-synonymous to Synonymous Substitution Ratio
Phytozome: Plant Genome Database
TAIR: The *Arabidopsis* Information Resource
Pfam: Protein Family Database
PlantTFDB: Plant Transcription Factor Database
PlantCARE: Plant Cis-acting Regulatory Element Database
miRBase: microRNA Database
ORF: Open Reading Frame
pI: Isoelectric Point
GRAVY: Grand Average of Hydropathicity
BP: Biological Process (GO term)
CC: Cellular Component (GO term)
MF: Molecular Function (GO term)
CREs: *Cis*-acting Regulatory Elements
*AtDCL*: *Arabidopsis thaliana* Dicer-like
*AtAGO*: *Arabidopsis thaliana* Argonaute
*AtRDR*: *Arabidopsis thaliana* RNA-dependent RNA Polymerase
MYA: Million Years Ago
TPM: Transcripts Per Million
